# Genetic history of Calabrian Greeks reveals ancient events and long term isolation in the Aspromonte area of Southern Italy

**DOI:** 10.1038/s41598-021-82591-9

**Published:** 2021-02-04

**Authors:** Stefania Sarno, Rosalba Petrilli, Paolo Abondio, Andrea De Giovanni, Alessio Boattini, Marco Sazzini, Sara De Fanti, Elisabetta Cilli, Graziella Ciani, Davide Gentilini, Davide Pettener, Giovanni Romeo, Cristina Giuliani, Donata Luiselli

**Affiliations:** 1 Department of Biological, Geological and Environmental Sciences, University of Bologna, Bologna, Italy; 2 Department of Cultural Heritage, University of Bologna, Ravenna, Italy; 3 Interdepartmental Centre Alma Mater Research Institute on Global Challenges and Climate Change, University of Bologna, Bologna, Italy; 4 Department of Brain and Behavioral Sciences, University of Pavia, Pavia, Italy; 5 Italian Auxologic Institute IRCCS, Cusano Milanino, Milan, Italy; 6 Medical Genetics Unit, Sant’Orsola-Malpighi University Hospital, Bologna, Italy; 7 European School of Genetic Medicine, Bologna, Italy

**Keywords:** Population genetics, Biological anthropology

## Abstract

Calabrian Greeks are an enigmatic population that have preserved and evolved a unique variety of language, *Greco*, survived in the isolated Aspromonte mountain area of Southern Italy. To understand their genetic ancestry and explore possible effects of geographic and cultural isolation, we genome-wide genotyped a large set of South Italian samples including both communities that still speak *Greco* nowadays and those that lost the use of this language earlier in time. Comparisons with modern and ancient populations highlighted ancient, long-lasting genetic links with Eastern Mediterranean and Caucasian/Near-Eastern groups as ancestral sources of Southern Italians. Our results suggest that the Aspromonte communities might be interpreted as genetically drifted remnants that departed from such ancient genetic background as a consequence of long-term isolation. Specific patterns of population structuring and higher levels of genetic drift were indeed observed in these populations, reflecting geographic isolation amplified by cultural differences in the groups that still conserve the *Greco* language. Isolation and drift also affected the current genetic differentiation at specific gene pathways, prompting for future genome-wide association studies aimed at exploring trait-related loci that have drifted up in frequency in these isolated groups.

## Introduction

The Italian Peninsula represents a key area of investigation to explore population and demographic processes that characterized the peopling history of Europe and the Mediterranean, and to reconstruct patterns of genetic diversity at different geographical scales^1^. The genomic variability among present-day Italians is indeed due to the multi-layered mosaic of pre-historical and historical processes of migration and admixture that interested the continent throughout time, but also to its valuable diversity and richness in terms of ecological complexity and cultural heterogeneity. The existence of distinct environmental pressures and climate conditions were proved to have forced different patterns of local adaptations between the Northern and Southern Italy, contributing to the observed genetic structure^2,3^. In the same way, geographic constraints or cultural factors (e.g. language, ethnicity, socio-economic structure) were suggested to have additionally influenced human population variability along the Peninsula, being responsible for different paths of isolation and differentiation or population mobility at more fine-grained local levels^4–6^.

The distinctive genomic variability of Italy has been largely investigated by using both uniparental markers^7–10^, autosomal SNP-chip data^2,11–13^ and, more recently, through one of the first study on whole genome sequencing^3^. In this context, the analysis of geographically isolated populations or of cultural enclaves and ethno-linguistic minorities can provide a simplified observatory for exploring population relationships within and among human groups. Indeed, the condition of isolation might have helped reducing the confounding effects of admixture^14,15^. Furthermore, the founding event and the limited external gene flow, ultimately resulting in smaller effective population size (Ne) and increased homozygosity and linkage disequilibrium (LD), may help the study of potentially trait-associated alleles found at higher frequency in these groups, thus making population isolates key models for genome-wide association studies^16–20^. In line with this viewpoint, some of the geographic and cultural isolates today settled in the Italian territory have long attracted the attention of population genetic studies based on both uniparental and autosomal markers^4,5,21–26^.

Recently, a genomic survey on patterns of ancient and recent admixture in Southern Italy and the Mediterranean^27^ brought specific interest on the enigmatic Calabrian Greek-speaking communities residing in the Aspromonte mountain area of Bovesia, in the territory of Reggio Calabria (Southern Italy). These ethno-linguistic groups represent extant Hellenic islands in Southern Italy that still preserve a unique variety of Greek, also known as *Greco* or *Calabrian Greek*. Linguistic studies have supplied interesting information about the possible origins of these communities, historically counterpoising two main antithetic hypotheses. The first hypothesis leads back to the Medieval period and suggests that this language might derive from the descendants of Byzantines who settled Southern Italy between the fifth and eleventh centuries^28–32^. The second hypothesis instead brings to the *Magna Graecia* colonization of Southern Italy in the eighth century BC to trace the origin of the language. Thereafter, the Greek of Calabria would have been uninterruptedly spoken during the centuries until the present, with local developments^33–35^. More recently, reconciling scenarios tried to mitigate the initial controversy between the “Hellenic” vs. “Byzantine” dichotomy, mostly in virtue of two considerations: (i) the impact of intense Greek-Romance linguistic contacts into a dynamic model of coexistence and cohabitation between Greek-speaking Latins and Latin-speaking Greeks; and (ii) the implications of archaic lexical elements shared with peripheral Greek dialects such as those of Cyprus and the Dodecanese, to assess the importance of linguistic contributes from the Greek of different periods^36,37^. The new moderated hypotheses in fact consider the Greek of *Magna Graecia* to have been survived in diglossia with Latin during the Roman Empire and then to have been rekindled in the Byzantine era and subsequent periods. Importantly, multiple strata which repeatedly brought waves of Greek speakers onto the Calabrian coasts were suggested to have overall contributed to the Greek heritage of the region^38^. Historically, it is known that the Greek presence in Calabria was continuous since ancient times and that the area of Greek-influence in Southern Italy was originally more largely extended with respect to the enclaves present nowadays^39^. In fact, the number of *Greco*-speaking people is rather limited at present and today the language survives mainly in few communities residing in the Aspromonte mountain area of Reggio Calabria^40,41^.

**Figure 1:**
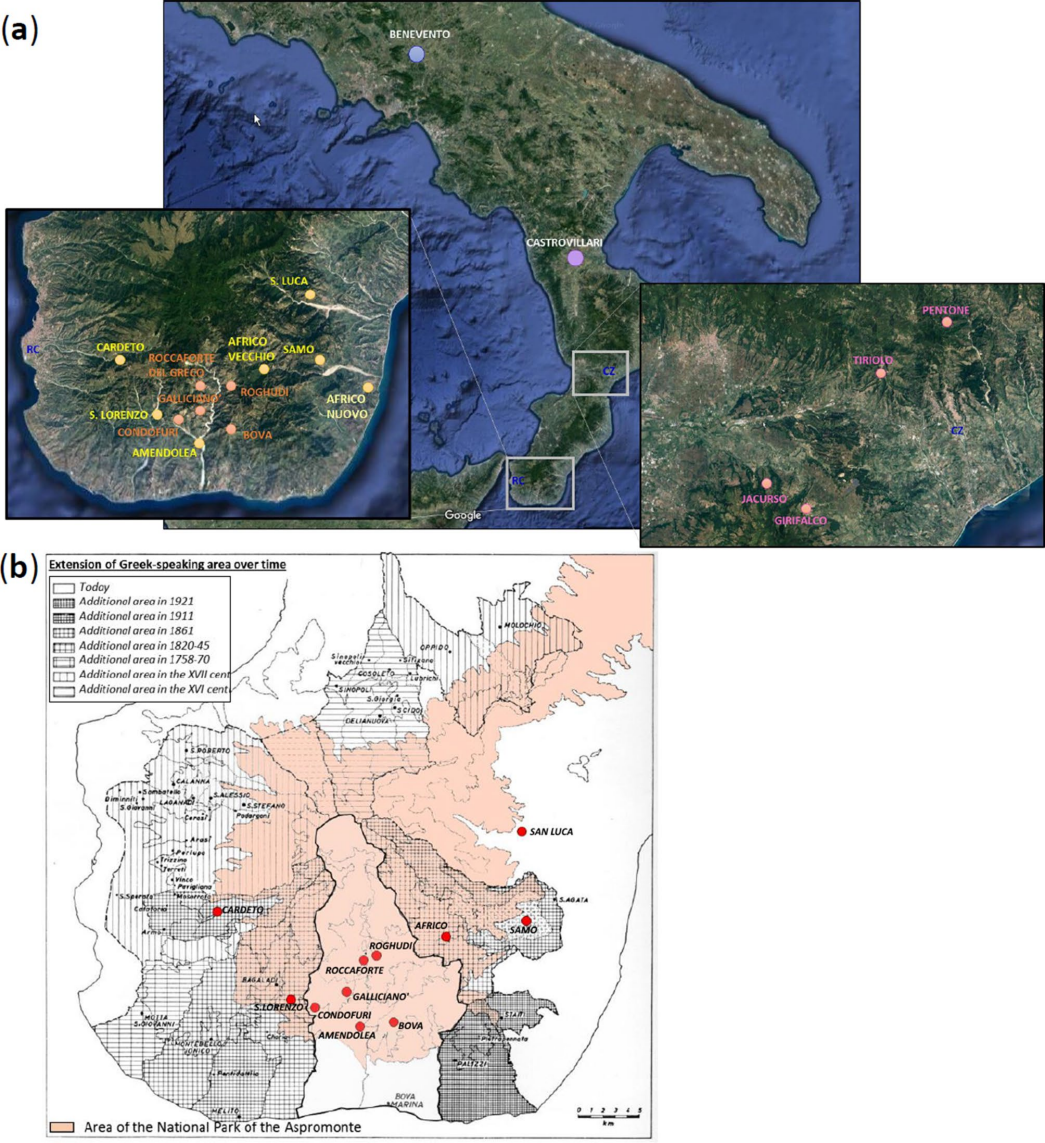
(**a**) Sampling map showing the approximate geographic location of analyzed populations. Sampling points are color-coded according to the province of origin: Benevento (*blue*); Castrovillari (*purple*); Catanzaro (*magenta*); previously collected samples from Reggio Calabria (*orange*); newly collected samples from Reggio Calabria (*gold*). The two enlarged boxes detail the sampling locations of villages in the province of Reggio Calabria (left) and in the province of Catanzaro (right), respectively. (**b**) Historical map showing the approximate extension of the National Park of the Aspromonte mountain area (in pink) as well as the range of the Greek-speaking area at different time periods as reported in the legend at the top-left. Geographical map has been generated with the package *RgoogleMaps [v. 1.4.1]*^92^ (URL: http://www.jstatsoft.org/v63/i04/) of the software R [v. 3.2.4] (https://www.r-project.org/).

Our previous characterization of Calabrian Greek communities for approximately 150,000 genome-wide SNPs with the Illumina GenoChip 2.0 DNA Ancestry chip suggested possible signs of genetic drift experienced by these groups^27^. In the present study, we further address issues of geographic and cultural isolation by using a higher in-depth level of analysis, which was achieved both by increasing the number of analyzed markers (720 K) and by genotyping also other communities from the same geographic area of Southern Italy (Fig. 1a). In particular, we significantly expanded the population samples from the Aspromonte mountain area by including six additional communities (Amendolea, Africo, San Lorenzo, Cardeto, Samo, San Luca) to the five already collected previously (Bova, Roghudi, Roccaforte del Greco, Gallicianò, Condofuri), therefore doubling the representativeness of the genetic structure of the area. The sampling strategy specifically covered both the communities that still preserve the *Greco* language as well as those from the same geographically isolated area of the Aspromonte that lost the use of this language earlier in time (Fig. 1b). This more extended sampling should therefore mirror the progressive restriction of the area of Greek-influence in the Southern Calabrian territory of Reggio Calabria. The groups from the Aspromonte mountain area were finally compared with newly-collected samples coming from a similar, but less isolated geographical context, which encompasses four villages from the Calabrian province of Catanzaro (Girifalco, Jacurso, Pentone, Tiriolo), as well as with ‘open’ (i.e. not isolated) Southern Italian groups from Castrovillari (Northern Calabria, Southern Italy) and Benevento (Campania, Southern Italy) (Fig. 1a). In this context, the aim of this study is to investigate the past population events and the local demographic factors that significantly contributed to the current genetic differentiation of Calabrian Greeks. In particular, by comparing their allelic architecture to the more general Southern Italian population we looked for the effect of geographic and cultural isolation on the detected genetic structure.

## Results

### Population structure of Southern Italy within the Euro-Mediterranean genetic landscape

**Figure 2:**
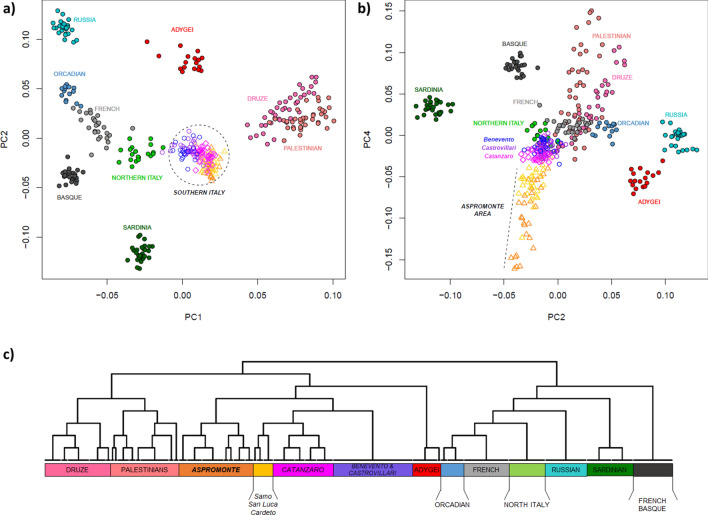
Principal component analysis and FineSTRUCTURE clustering analysis performed on the extended comparison dataset of modern populations. Scatterplots of the (**a**) first vs. second and (**b**) second vs. fourth PCs are reported on the top of the plot. Individuals are color-coded based on their geographic location. Newly analyzed populations from Southern Italy and, more specifically, those from the Aspromonte mountain area are labelled in the first and second plot respectively. (**c**) FineSTRUCTURE hierarchical clustering dendrogram calculated between the pairs of modern individuals included in the extended comparison dataset. The 13 detected clusters, here highlighted with different colors, are highly concordant with population labels. For the detailed annotation of individuals inside of each cluster see Supplementary Figure S3.

In order to set the observed genetic variability into a wider context, a PCA was firstly performed by comparing our newly analyzed Southern Italian populations to Mediterranean and European groups extracted from the HGDP (Suppl. Table S1). The plot of the first two principal components (Fig. 2a) recapitulates well-known geographic patterns of genetic variation commonly observed in the Euro-Mediterranean area^42,43^. In fact, the PC1, extending from the Levantine groups of Palestinians and Druze to Russians, Orcadians and French, identifies a South-East to North-West axis of genetic structuring. On the other hand, PC2 emphasizes the renowned outlying position of Sardinia within the European genetic landscape^44,45^. Populations from the Italian Peninsula reflect the known latitudinal cline of genetic differentiation between North and South Italy^2,3^, with the former closer to Western Europe and the latter projecting towards the Near East. Interestingly, populations from the Aspromonte area depart from the other Southern Italian groups when PC4 is considered (Fig. 2b). Accordingly, ADMIXTURE analysis identifies the main European-, Near Eastern- and Sardinian-like genetic ancestries, to which an Aspromonte-specific component is added for higher values of K (Suppl. Figure S1).

F3-statistic was then used to formally test each Southern Italian group as target of admixture using all the other comparison population pairs as putative parental sources. Statistically significant results (i.e. Z-scores < −3) for a mixture between Sardinian and Caucasian or between Near-Eastern and continental European sources were obtained for the populations of Benevento, Castrovillari and Catanzaro (Suppl. Table S2). Consistently with these results, the maximum likelihood tree reconstructed with Treemix (Suppl. Figure S2) locates all the Southern Italian groups in an intermediate position between Caucasian and Near Eastern populations on one hand, and a continental European cluster encompassing Orcadian, Russian, French, Basque and North Italian populations together with Sardinians in a rooted position, on the other hand. When allowing for admixture, TreeMix optimized the fit of the data to the tree by adding two migration edges between populations, specifically between Russians and Adygei, and between Sardinian and French_Basque. Interestingly, adding further admixture events (from m = 3 through m = 6) increased the rate of explained variation in the data by the tree models, revealing migration edges from Caucasus (Adygei) or from a Caucasian/Near-Eastern root to the Southern Italian populations of Benevento, Castrovillari and Catanzaro. This happens each time they were included within the continental European group instead of splitting out before it (Suppl. Figure S2), thus providing further support for genetic links between Southern Italy and the Near-East/Caucasus as consistently observed by f3-admixture tests. In this context, it is noteworthy that population samples from the Aspromonte area do not show evidence of gene flow from any other group (Suppl. Table S2) and in the Treemix phylogeny appear instead located in a basal position with respect to all the other Southern Italian populations (Suppl. Figure S2). At the same time, they also show longer branch length, thus signaling possible evidence of more ancient isolation and drift effects.

To empirically evaluate finer-scale patterns of structuring, we used the haplotype-based approach implemented in CHROMOPAINTER/FineSTRUCTURE to define clusters of genetically homogeneous individuals. Overall, the clusters recognized by FineSTRUCTURE (Fig. 2c) largely match with local population groups and summarize the patterns of genetic relationships consistently observed in genotype-based analyses. In fact, populations from Southern Italy globally form a clade that is related to the Caucasian Adygei group and, at a more basal level, to the Near Eastern clusters formed by Palestinians and Druze, respectively. With a more specific focus, it is worth noting how FineSTRUCTURE virtually reconnects almost all the populations from the Aspromonte mountain area to a specific cluster, which splits up as a separated group with respect to all the other Southern Italian populations (Fig. 2c, Suppl. Figure S3). Within this “*Aspromonte*” (*ASPR*) cluster, the *Greco*-speaking communities from Roghudi, Roccaforte del Greco, Condofuri and Gallicianò form a further sub-group that exhibits more remarkable signals of genetic drift (Suppl. Figure S4). On the other hand, samples from Cardeto, Samo and San Luca fall outside from this “*Aspromonte*” (*ASPR*) cluster and appear instead grouped to the other Southern Italian populations, namely to the cluster of villages from the province of Catanzaro (*CZ*) and then to a cluster indifferently encompassing all the individuals from both Benevento and Castrovillari (*BN* + *CS*) (Fig. 2c, Suppl. Figure S3).

### Local patterns of isolation and genetic differentiation within Southern Italy

With the aim of directly exploring local patterns of genetic differentiation, we focused more specifically on the genetic structure observed within Southern Italy. Consistently with the global analysis, the PCA applied exclusively on our Southern Italian “local” dataset replicates the distinctiveness observed for the Aspromonte group (Suppl. Figure S5a). Most of the populations from that area indeed depart from the rest of Southern Italy along the first PC, by forming a scattered pattern in which the communities that still preserve the *Greco* language appear as the most differentiated (i.e. occupying more peripheral positions in the PCA plot). Similarly, ADMIXTURE results for the best value of K = 2 (Suppl. Figure S5b) identify an ancestral genetic component which is maximized in almost all the current *Greco*-speaking communities (100% Roghudi and Gallicianò, 88% Roccaforte Del Greco, 73% Condofuri) and accounts for 30–60% of ancestry also in the core of the other communities from the same *Aspromonte* group.

**Figure 3:**
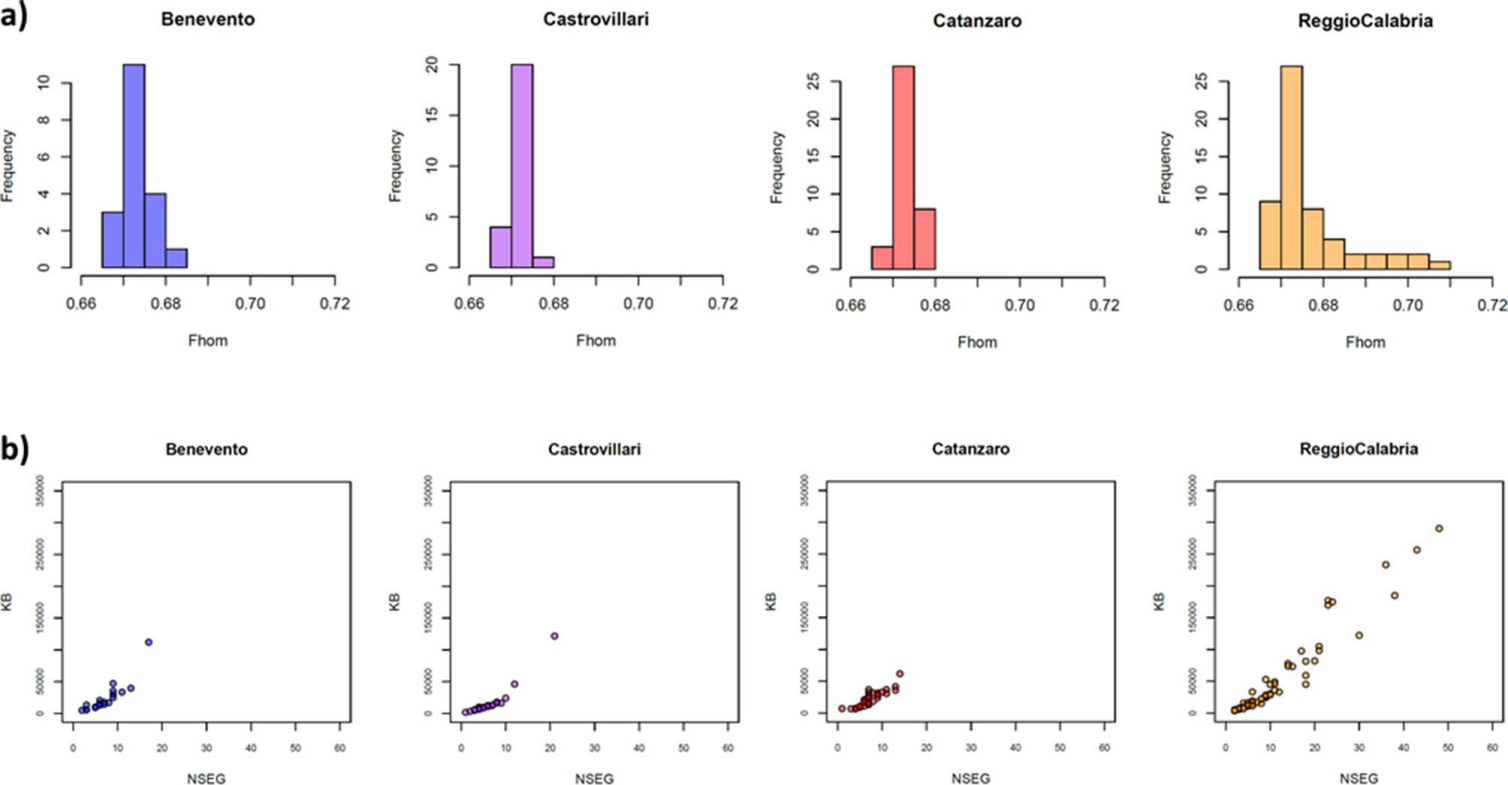
Intra-population patterns of genetic variation in the newly analyzed Southern Italian populations. (**a**) Distribution of genome-wide homozygosity index *Fhom* and (**b**) total length of ROHs (KB) plotted against number of ROHs (NSEG). Color-code as follow: Benevento (*blue*), Castrovillari (*purple*), Catanzaro (*magenta*) and Aspromonte area of Reggio Calabria (*orange*).

To formally test signals of isolation and drift, we analyzed patterns of within-population genetic variation by calculating the inbreeding coefficient (*Fin*) and the genome-wide homozygosity (*Fhom*) indexes, and by analyzing the number and the extension of genomic runs of homozygosity (ROH). Both *Fin* and *Fhom* values are averagely higher for the populations from the Aspromonte area compared to the rest of Southern Italy (Suppl. Table S3), and accordingly the distribution of genome-wide homozygosity (*Fhom*) shows higher variance in inbreeding for the Aspromonte group than for the other Southern Italian populations (Fig. 3a), thus reflecting higher isolation patterns differentiating these communities. Similarly, a much higher number (NSEG) and length (KB) of ROH has been observed on average for the Aspromonte samples from Reggio Calabria, with respect to the tendency to lower number and length of homozygous segments exhibited by the other Southern Italian populations (Fig. 3b).

Since levels of isolation correlate also with the degree of relatedness between and within groups^46^, we further estimated the extent of genome shared identically by descent (IBD) at different classes of length using the *fastIBD* pipeline implemented in the BEAGLE software. Overall, patterns of genetic relatedness across populations reveal higher within-population compared to between-population sharing for longer bin classes. In particular, for bins ≥ 27 cM almost all the connections are within-population and the few links between-populations appear almost exclusively limited to the communities belonging to the *ASPR* cluster, consistently with these groups being more closely related to each other. For shorter classes of length among-population connections then extend to all the rest of Southern Italy (Suppl. Figure S6).

To further explore signals of genetic drift and population differentiation and to link this peculiar genetic background with biological functions, we finally used the Southern Italian genetic clusters identified by FineSTRUCTURE to detect loci that may have drifted up in frequency in the isolated Aspromonte group. In particular, we compared the allele frequencies of all variants within the populations belonging to the *ASPR* cluster against their respective frequencies in the rest of South Italy. More precisely, as suggested by FineSTRUCTURE results (Fig. 2c, Suppl. Figure S3), we considered both the *Benevento* + *Castrovillari* (*BN* + *CS*) cluster and the one grouping individuals from *Catanzaro* (*CZ*) as representative of the “not-isolated” Southern Italian background. We then computed pairwise FST values between the above-mentioned three clusters (i.e. *ASPR, CZ* and *BN* + *CS*) for all the 621,755 SNPs included in our “local” dataset. Finally, we retained the 797 loci scoring in the top 1% of FST distribution in both *ASPR* vs. *CZ* and *ASPR* vs. *BN* + *CS* comparisons (Suppl. Figure S7, Suppl. Table S4). Enrichment analysis on the list of corresponding top genes (Suppl. Table S5) shows that the most significantly enriched Gene Ontology (GO) terms are associated with processes of “*nervous system development*” and with “*neuron part*”, “*cell periphery*” and “*plasma membrane*” of the cellular components (Suppl. Table S6).

### Ancient genetic heritage of Southern Italian populations

Since present-day patterns of genetic variation reflect both local dynamics of differentiation and the ancestral population history, in order to provide a temporal overview on the ancestral genetic legacy of analyzed Southern Italian groups we finally compared the genetic landscape defined by modern populations with a large panel of ancient DNA samples extracted from the literature and timewise spanning from the Mesolithic to the Iron Age (Suppl. Table S7).

Consistently with previous results^3,27^, the PCA performed by projecting ancient samples onto the modern genetic variation reveals specific patterns of population relationships (Suppl. Figure S8). In fact, all the Southern Italian groups, besides showing a general high affinity with Anatolian and European Neolithic farmers, cluster also closely with the Chalcolithic and Bronze Age samples from Anatolian and Aegean (Minoan and Mycenaean) populations. Differences in affinity patterns were formally tested with the *outgroup-f3* statistic measuring the extent of shared drift between modern Italian groups and the main ancient genetic components represented by Western European Hunter-Gatherers (WHG), Eastern European Hunter-Gatherers (EHG), Caucasian Hunter-Gatherers (CHG), Anatolian Neolithic farmers (AN) and Pontic-Steppe Yamnaya (EMBA). Overall Sardinia shows the highest levels of shared drift with samples of Neolithic-related ancestry compared to Northern and Southern Italy. Both Sardinians and Northern Italians show higher affinity to WHG than Southern Italians, who instead appear more affected by CHG-related groups. On the other hand, Yamnaya Steppe and EHG share more affinity to North Italy than to both Southern Italians and Sardinians (Suppl. Figure S9). In addition, *qpGraph-*based phylogenies consistently recapitulate the observed genetic patterns, with Sardinians showing a good fit to a two-way mixture model between populations representing Early European Farmers and West European Hunter-Gatherers (Suppl. Figure S10a), and North Italy instead achieving a successful fit to a graph model with an additional admixture event from an EHG-related lineage (Suppl. Figure S10b). Interestingly, when fitting present-day Southern Italian populations into the tested *qpGraph* models we find them compatible with an additional contribute that, differently from Northern Italy, does not originate from an EHG-related source but instead from a CHG-related lineage (Suppl. Figure S10c). This fits to the data in the sense that there are no f-statistics more than |Z| > 3 different between model and expectation.

**Figure 4:**
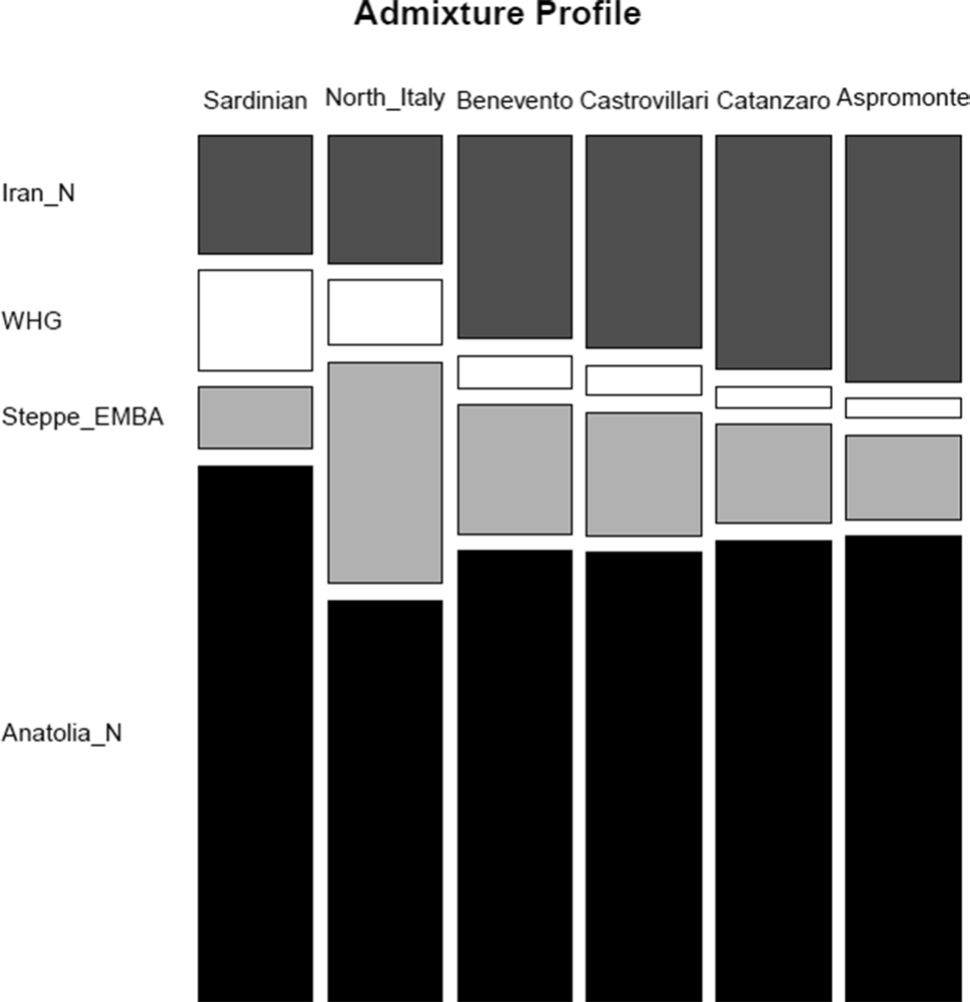
Mosaic plot of admixture ancestry profiles as inferred by *qpAdm*. Admixture profiles of Italian population groups included in the modern extended dataset have been tested using a four-population model including CHG/Iran_Neolithic, WHG, Steppe_EMBA and Anatolian_Neolithic as putative ancient source groups. Specific details about inferred ancestry proportions and relative levels of significance are reported in the Supplementary Table S8.

Finally, to better characterize the ancestral composition of Southern Italian populations, we inferred their mixture proportions with respect to a four-population model of admixture including all the above-mentioned WHG, Neolithic, CHG/Iran_N and Steppe-related main sources, using *qpAdm*. All Italian populations were successfully modeled as characterized by a relatively high amount of Anatolian Neolithic ancestry, with the major contribution observed in Sardinians (Fig. 4, Suppl. Table S8). The remaining ancestries were assigned to a lower WHG contribution and to differential influences of Steppe_EMBA and CHG/Iran_N in the profiles of Northern and Southern Italians, respectively (Fig. 4, Suppl. Table S8). In fact, while Steppe ancestry is greater in North Italy (~ 27%), the Iran_N/CHG-related source is more present in South Italy with the highest values (~ 29%) observed in the populations from the Aspromonte area.

## Discussion

The origins of the *Greco*-speaking communities today settled in the Aspromonte mountain area of Reggio Calabria (Southern Italy) have been largely debated from a linguistic point of view. The first hypotheses defending the continuity of the language from the *Magna Graecia* or its Byzantine origin have been more recently reconciled into composite scenarios, in which the importance of longstanding contacts and multiple contributions from different periods have been reevaluated. As a matter of fact, the territory corresponding to present-day Calabria has been inhabited since prehistoric times and its centrality in the Mediterranean Sea is attested by the presence of artefacts from the most important Neolithic cultures of Southern Italy. Furthermore, its richness in mineral deposits testifies exchanges with the Aegean and Asia Minor civilizations that must have been intense during Metal Ages^47,48^. Accordingly, the extent of population and cultural interactions between South Italy and the southern part of the Balkan Peninsula including Greece, Crete and the Aegean islands has been confirmed by the presence of Mediterranean genetic links between these regions tracing back to Neolithic and post-Neolithic times^2,3,27^.

In this study, we analyzed the genetic variability of Calabrian *Greco*-speaking groups in the context of the local Southern Italian genetic landscape and with respect to the temporal and spatial structuring of the Euro-Mediterranean genetic variation, with the aim to infer the main demographic processes that shaped the genetic heritage of these populations. To this end, we collected a large set of samples representative of the communities settled in the Aspromonte mountain area, including both those that conserved *Greco* up to the present and the ones that lost the use of this language earlier in time. Then, we compared them against the genomic patterns observed for other not-isolated populations from Southern Italy as well as to a wider reference panel composed of both modern and ancient samples.

Overall, population structure analyses agree with previous studies and generally confirm the presence of strong genetic links between Southern Italy and the Caucasus/Middle-East^3,27^. Inferences of ADMIXTURE proportions indeed revealed the ancestry of present-day Southern Italian populations, regardless of their linguistic affiliation, to be composed mainly by Sardinian-like and South-Eastern Mediterranean genetic components, with a negligible contribution from a continental Eastern European ancestry instead higher in Northern Italy (Suppl. Figure S1). Accordingly, *f3*-tests (Suppl. Table S2) fitted a scenario involving mixtures between Sardinia and Caucasus or between Near Eastern and continental European-related ancestries to account for the genetic composition of present-day Southern Italian groups, also showing ancestral genetic connections with a Caucasus or a Caucasus/Near-Eastern branch in the Treemix phylogeny (Suppl. Figure S2).

In this context, both global PCA and ADMIXTURE analyses revealed the genetic proximity of the Aspromonte communities to the other populations of Southern Italy (Fig. 2, Suppl. Figure S1), showing at the same time traces of differentiation. Overall, the analyses of intra-population diversity, measuring both the number and the total length of homozygous genotypes (Fig. 3, Suppl. Table S3) as well as the extent of genome-wide IBD-sharing (Suppl. Figure S6), indeed confirmed higher levels of genetic isolation commonly experienced by the Aspromonte populations, when compared to the other neighboring Southern Italian groups. Furthermore, at a local level the Aspromonte communities departed from the South Italian genetic background, with those more significantly isolated both geographically and culturally occupying the most peripheral positions in the PCA plot and also exhibiting a private genetic component, which indeed reaches the highest frequencies in the Aspromonte groups still speaking *Greco* (Suppl. Figure S5). Accordingly, within the *Aspromonte (ASPR)* specific cluster identified by FineSTRUCTURE (Fig. 2, Suppl. Figure S3) the “chunk-length” matrix of haplotypes shared between pairs of individuals, specifically pinpointed the currently *Greco*-speaking communities as the ones signaling higher levels of drift (i.e. the lowest proportion of haplotype “copying” with other groups, Suppl. Figure S4), thus reflecting patterns of geographic isolation in the Aspromonte area further amplified by cultural differences in the groups that conserved the *Greco* language.

On the whole, the observed patterns of variation seem therefore to confirm the presence of ancient genetic links between Southern Italy and the South-Eastern Mediterranean populations of Caucasus and the Near East, with the groups from the Aspromonte mountain area—and particularly those that still preserve the *Greco* language nowadays—that departed from this shared genetic background as a consequence of isolation phenomena.

Previous surveys on the ancient genetic legacy of Southern Italy pointed to genetic contributions linking Southern Italy and Mediterranean Greek islands with Anatolia and the Caucasus tracing back to migratory events occurred during the Neolithic and the Bronze Age, in which the Mediterranean served as a preferential crossroad^3,13,27^. In particular, while the expansion of Anatolian Neolithic farmers significantly impacted all the Peninsula, differential Bronze-Age contributions were observed for Southern Italy with respect to Northern Italian populations. Bronze Age influences in the gene pool of Southern Italians have been in fact associated to a non-steppe Caucasian-related ancestry carried along the Mediterranean shores at the same time, but independently from the Pontic-Caspian Steppe migrations that occurred through Continental Europe. Consistently with this viewpoint, genetic analyses performed by comparing our modern populations with the main ancient ancestral sources have displayed the clustering of analysed Southern Italian groups with Neolithic and Bronze Age samples from Anatolian, Aegean Minoan and Mycenaean populations, as opposed to the affinity of Northern Italy with Late-Neolithic and Bronze-Age samples from continental Europe (Suppl. Figure S8). Accordingly, both *f3-outgroup*, *qpGraph* and *qpAdmixture* analyses (Fig. 4, Suppl. Figure S9, Suppl. Figure S10) revealed influences related to a Steppe ancestry in the Northern Italian groups, instead paralleled in Southern Italy by an analogous Caucasian-related contribution from a non-Steppe CHG/Iran_N source. Importantly, the same ancestral sources are equally shared both by the present-day “open” (i.e. not-isolated) Southern Italian populations of Benevento, Castrovillari and Catanzaro, as well as by the geographically and linguistically-isolated communities of the Aspromonte mountain area (Fig. 4, Suppl. Table S8), thus signaling a common genetic background that possibly predates the linguistic hypotheses originally suggested about the times of formation of the *Greco* language in Southern Italy. Accordingly, we hypothesize that the genetic continuity between Southern Italian populations and the other Mediterranean groups may date back to these Neolithic and post-Neolithic events and may have been subsequently maintained and in some cases reinforced by continuous and overlapping gene flows following similar paths of diffusion and interaction between populations, among which the migrations of Greek-speaking people during the classical era (*Magna Graecia*) and/or in Byzantine and subsequent times. Therefore, the observed patterns could be linked to a tendency to mobility that has always characterized these populations, resulting in continuous cultural and genetic exchanges over time. That being so, the Calabrian Greek ethno-linguistic minorities of Southern Italy may be interpreted as the remnants of a wider area of Greek influence, that by virtue of their geographic isolation have preserved and evolved a unique variety of Greek which has survived through centuries in the mountains of the Aspromonte area. At this respect, the communities showing higher signatures of genetic isolation (Roghudi, Gallicianò, Condofuri and Roccaforte del Greco; Suppl. Figure S4, Suppl. Figure S5) are also the ones located in the more impervious areas of the Aspromonte, at the same time still conserving a certain number of *Greco* speakers (Suppl. Table S1)^40,41^.

Incorporating in future studies the information provided by whole genome sequence data will be an additional value to comprehensively understand the interplaying impact of complex demographic history and evolutionary processes. Recent studies (e.g.^49^) have made efforts to identify loci or regions of the genome evolving in truly neutral vs. non-neutral manner to perform demographic inferences based on whole-sequencing data, also stressing how a-priori assumptions on the neutrality of great part of the genome may bias some resultant inferences (see also^50,51^). Therefore, even if the limited temporal depth and relatively micro-geographical setting of the present study should in some way prevent relevant biases, future researches in these directions may integrate and be compared to the present work in order to obtain more accurate demographic inferences.

Besides the importance in population history, ethnogenesis and linguistic variation, demographic processes of isolation might have also affected the genetic composition of present-day groups inhabiting these areas of Southern Italy. In fact, the GO analysis showed peculiar biological function of genes related to neurological pathways with higher level of differentiation in the Calabrian area (Suppl. Table S6). Recent studies on hereditary neurodegenerative disorders such as Alzheimer’s, Frontotemporal Dementia and Parkinson diseases in Southern Italy were carried out and highlighted that certain areas of the Calabrian region are characterized by low genetic heterogeneity and high levels of consanguinity due to the geographic isolation over the centuries^52–58^. The observation of recurrent mutations and haplotypes in isolated populations with high rates of consanguinity might be potentially informative for the study of hereditary diseases. Overall, these data more generally remark the importance of population isolates in genetic studies. In fact, due to isolation and drift, coupled with the effects of smaller Ne and higher levels of consanguinity, isolated populations may have modified their genetic architecture through the random amplification or loss of certain genetic variants, thus allowing the study of the role of loci found at higher frequency in these groups. In this sense, future studies including also phenotypic data could be of extreme value to understand the role of trait-associated variants on health status as recently demonstrated by research efforts that have linked population genetics and medical genetics (e.g.^59^).

## Materials and methods

### Population samples

In this study, we collected and analyzed a total of 149 Southern Italian individuals belonging to 11 villages from the Aspromonte mountain area of Reggio Calabria (Southern Calabria), 4 villages from the province of Catanzaro (Central Calabria), and to population samples from the provinces of Cosenza (Northern Calabria) and Benevento (Campania) (Fig. 1, Suppl. Table S1).

Saliva samples were collected with the Oragene-DNA Self Collection Kit OG-500 (DNA Genotek, Ottawa, Ontario, Canada) from unrelated volunteers, by focusing on subjects with a local genetic ancestry over at least three generations in their respective communities of origin, which were also surveyed for language affiliation.

### Ethics statement

All donors provided a written informed consent to data treatment and project objectives, and all the procedures concerning this population genetics study was approved by the Bioethic Committee of the University of Bologna on 08/04/2013. The study was designed and conducted in agreement with relevant guidelines and regulations according to the ethical principles for research involving human subjects stated by the WMA Declaration of Helsinki.

### Genotyping and quality filtering

Genomic DNA was purified from Oragene-DNA collection kits following manufacturer’s recommendations and quantified with the Qubit dsDNA BR Assay Kit (Life Technologies, Carlsbad, CA, USA). DNA samples were then genotyped for the 713,014 SNPs implemented in the HumanOmniExpress BeadChip (Illumina, San Diego, CA, USA), by using the facilities available at the Center for Biomedical Research & Technologies of the Italian Auxologic Institute (Milan, Italy).

Genotyping results were filtered using the PLINK software 1.9^60^ after having excluded SNPs on the sex chromosomes. We removed all individuals with a genotyping success rate lower than 92%, variants with missing call rates exceeding 2%, SNPs with a minor allele frequency (MAF) lower than 1%, and markers showing significant deviations from the Hardy–Weinberg equilibrium. In addition, we estimated the degree of identity-by-descent (IBD) sharing and excluded one individual for each pair of samples with a kinship coefficient (PiHat) higher than 12.5%.

After filtering procedures, we obtained a final “local” dataset composed by 141 individuals typed for 621,755 autosomal SNPs markers. The dataset was thinned for genotype-based analyses by removing SNPs in LD (r2 > 0.1) within a sliding window of 50 SNPs advanced by 10 SNPs at the time (PLINK option *--indep-pairwise 50 10 0.1*), obtaining a “pruned local” dataset consisting of 64,147 SNPs.

### Comparison datasets

In order to frame the variability of analyzed populations into the Euro-Mediterranean genetic landscape, we merged our Southern Italian “local” dataset with publicly available genome-wide data from Europe, Near East and the Caucasus, extracted from the Human Genome Diversity Project (HGDP)^61^. The same QC described above for the local population set were performed on the reference dataset, further removing ambiguous A/T and C/G polymorphisms to avoid strand-flipping issues during merging procedure. After merging, we obtained a “modern extended” dataset including 238 additional individuals from 10 Euro-Mediterranean comparison populations (Suppl. Table S1) and a common set of 337,711 SNPs (59,124 SNPs after pruning for *--indep-pairwise 50 10 0.1* as above).

To test temporal patterns of genetic relationships, we finally merged the “modern extended” dataset with genomic data for 1059 ancient samples (Suppl. Table S7) extracted from the literature^62–72^ and genotyped on the 1240 K panel (V37.2.1240K, https://reich.hms.harvard.edu/), finally obtaining a common “modern-plus-ancient” dataset of 326,832 SNPs. For the genotype-based analyses involving also ancient samples we applied a LD-pruning procedure by excluding one SNP for each pair of loci showing r2 values higher than 0.4 within a 200‐SNPs window, sliding 25 loci at the time (PLINK option *--indep-pairwise 200 25 0.4*), for a total of 286,656 SNPs left after pruning.

### Population structure and admixture analyses

Principal Component Analysis (PCA) was performed on the “local” and “extended” datasets including modern populations by using the *smartpca* function implemented in the EIGENSOFT package^73^. Ancient samples were then projected onto the PCA space obtained from the modern populations by using the *lsqproject* = *YES* function.

Inferences of ancestry proportions for the modern groups were estimated with the ADMIXTURE software^74^, by testing hypothetical ancestral populations (K) from 2 through 10. We performed ten independent runs with different random seeds for each given K and used those with the highest log-likelihood values for the final plot. Cross‐validation (CV) errors were also calculated for each run with the aim of identifying the number of K showing the best fit to the data.

Genetic relationships and gene-flow patterns between modern populations were explored using the Treemix v1.12 software^75^. We run Treemix including a North-African population (*Mozabites*) as root to build a phylogenetic tree without allowing for migration, and then we tested an increasing number of migratory events from m = 1 to m = 6.

To formally assess affinity patterns between modern groups and ancient individuals we computed *outgroup-f3* statistics in the form of *f3(YRI; Modern, Ancient)*, by using the *qp3pop* function implemented in the ADMIXTOOLS package^76,77^. Furthermore, to test models of phylogenetic relationships between present-day and ancestral populations, we applied the modeling approach implemented in the *qpGraph* software of the ADMIXTOOLS v3.0 package^77^, relying on defined topologies with ancient West Eurasian groups^62,78^. In order to keep the models simple, we started with small, well-understood subgraph by adding additional sources one at a time and testing at each subsequent step of the analysis the fitting of present-day populations as being explained by a mixture of two possible ancestral populations. In particular, by starting with a skeleton tree including Mbuti, WHG and MA1, we consequently grafted onto this basic phylogeny additional putative ancient sources representative of Early farmers, Eastern Hunter Gatherers (EHG) and Caucasian Hunter Gatherers (CHG)^78^. Then, we tried to explore the fitting of the analyzed modern Italian populations (particularly Southern Italian ones, compared to North Italy and Sardinians) into the progressively considered *qpGraph*-based phylogenies, evaluating the fits to the models based on the maximum |Z|-score comparing predicted and observed values. Finally, to better characterize the ancestral composition of analyzed modern Italian groups, we exploited the modeling approach implemented in *qpAdmix*^63^ to quantitatively estimate the admixture profile of each modern *Test* population using a four-population model of ancient ancestral *Source* groups (“Left” populations) with respect to a specific set of *Outgroups* (“Right” populations). In details, we first checked whether the considered “Right” and “Left” populations were significantly distinguishable by using *qpWave* and the following set of outgroups (Ust_Ishim, Kostenki14, MA1, GoyetQ116-1, ElMiron, Vestonice, Villabruna, EHG, Levant_N, Natufian, Mota) as defined previously^79^. Then, by using the same set of outgroups, we performed *qpAdm* runs to establish if the admixture profile of each target modern population was consisted with the selected set of ancient ancestral sources (i.e. WHG, CHG/Iran_N, Anatolian_Neolithic, Steppe_EMBA), inferring their relative admixture proportions. We considered a P-value threshold of 0.01 to assess the significance of tested models.

### Haplotype-based analysis of fine-scale structuring

To explore fine-grained patterns of population structure and define genetic clusters of homogeneous individuals, we exploited the haplotype-based approach implemented in CHROMOPAINTERv2/ fineSTRUCTURE^80^. Samples were phased using SHAPEIT^81^ by applying default parameters and using HapMap phase 3 recombination maps. We run CHROMOPAINTER analysis on the 379 individuals of the “unpruned modern extended” dataset, by initially estimating the switch and mutation/emission rates on a subset of four chromosomes {4, 10, 15, 22} using 10 steps of Expectation–Maximisation (E-M). Then, we averaged the inferred values across these chromosomes, weighting by the number of markers and individuals, and we exploited the obtained parameters to re-run CHROMOPAINTER on all chromosomes using each individual both as “donor” and “recipient”. The obtained matrix of shared haplotype “chunk-counts”, combined across the 22 autosomes, was submitted to the fineSTRUCTURE clustering algorithm version fs2.1^80^. We ran fineSTRUCTURE by setting 3,000,000 “burn-in” MCMC iterations, followed by 2,000,000 additional iterations where the inferred clustering patterns were sampled every 10,000 runs. Finally, we set 1,000,000 additional hill-climbing steps to improve posterior probability and merge clusters in a step-wise fashion until reaching the final configuration tree.

### Analyses of genetic isolation and population differentiation

To explore patterns of within population genetic variation, we calculated the number and extension of ROH segments^82,83^ and the *Fin* and *Fhom* indexes by using respectively the *--homozyg* and *--het* functions of PLINK software as described previously^59^.

Furthermore, we estimated patterns of IBD sharing within and among Southern Italian populations using the *fastIBD* method implemented in the BEAGLE 3.3 software^84^. Data were phased with SHAPEIT^81^ as specified above and *fastIBD* was run ten times for each chromosome using different random seeds. IBD blocks were called by post-processing the obtained results with the *‘plus-process-fibd.py’* pipeline^85^, setting the *fastIBD* threshold to 1e-10 and considering only blocks longer than 1 cM. We then explored the distribution of segments shared IBD between pairs of individuals both within- and among-populations for different bins of length (in cM) to approximate different degrees of relatedness^86^.

Finally, to examine possible signals of genetic differentiation in the isolated groups of Aspromonte area with respect to the more general Southern Italian population, we compared allele frequencies between the population clusters identified by fineSTRUCTURE, computing single locus Weir and Cockerham Fst for each of the 621,755 SNPs included in the high-density “local” dataset. More precisely, we retained the top 1% of markers in the Fst distribution that differentiate the *Aspromonte* cluster from both *Catanzaro* and *Benevento* + *Castrovillari* comparison clusters. The list of genes encompassing the detected top 1% most differentiating SNPs was compared to the reference list of all genes covered by the Illumina HumanOmniExpress BeadChip and submitted to an Enrichment Analysis using the PANTHER Gene Ontology (GO) tool^87,88^ with the aim to pinpoint the most relevant pathways involved in the observed differentiation. Information about chromosome location, start and end positions of each gene, as well as its approved name and alternative nomenclature was built by cross-checking the Ensembl database gene list for the human reference genome hg37.p13 (Ensembl GRCh37 human archive, release 100) and the HUGO Gene Nomenclature Committee resource (http://www.genenames.org, March 2020), with data recovered through the BioMart data mining tool in both cases^89–91^.

## Supplementary Information

Supplementary Information 1.

Supplementary Information 2.

## References

[1] Sazzini M, Sarno S, Luiselli D, Goffredo S, Dubinsky Z (2014). The Mediterranean human population: an anthropological genetics perspective. The Mediterranean Sea: Its History and Present Challenges.

[2] Sazzini M (2016). Complex interplay between neutral and adaptive evolution shaped differential genomic background and disease susceptibility along the Italian peninsula. Sci. Rep..

[3] Sazzini M (2020). Genomic history of the Italian population recapitulates key evolutionary dynamics of both Continental and Southern Europeans. BMC. Biol..

[4] Destro Bisol G (2008). Italian isolates today: Geographic and linguistic factors shaping human biodiversity. J. Anthropol. Sci..

[5] Coia V (2013). Demographic histories, isolation and social factors as determinants of the genetic structure of Alpine linguistic groups. PLoS ONE.

[6] Boattini A (2015). Traces of medieval migrations in a socially stratified population from Northern Italy. Evidence from uniparental markers and deep-rooted pedigrees. Heredity.

[7] Turchi C (2008). Italian mitochondrial DNA database: Results of a collaborative exercise and proficiency testing. Int. J. Legal. Med..

[8] Capelli C (2007). Y chromosome genetic variation in the Italian peninsula is clinal and supports an admixture model for the Mesolithic-Neolithic encounter. Mol. Phylogenet. Evol..

[9] Brisighelli F (2012). Uniparental markers of contemporary Italian population reveals details on its pre-Roman heritage. PLoS ONE.

[10] Boattini A (2013). Uniparental markers in Italy reveal a sex-biased genetic structure and different historical strata. PLoS ONE.

[11] Di Gaetano C (2012). An overview of the genetic structure within the Italian population from genome-wide data. PLoS ONE.

[12] Fiorito G (2016). The Italian genome reflects the history of Europe and the Mediterranean basin. Eur. J. Hum. Genet..

[13] Raveane A (2019). Population structure of modern-day Italians reveals patterns of ancient and archaic ancestries in Southern Europe. Sci. Adv..

[14] Ayub Q (2015). The Kalash genetic isolate: Ancient divergence, drift, and selection. Am. J. Hum. Genet..

[15] Cilli E (2019). The genetic legacy of the Yaghnobis: A witness of an ancient Eurasian ancestry in the historically reshuffled central Asian gene pool. Am. J. Phys. Anthropol..

[16] Peltonen L, Palotie A, Lange K (2000). Use of population isolates for mapping complex traits. Nat. Rev. Genet..

[17] Service S (2006). Magnitude and distribution of linkage disequilibrium in population isolates and implications for genome-wide association studies. Nat. Genet..

[18] Kristiansson K, Naukkarinen J, Peltonen L (2008). Isolated populations and complex disease gene identification. Genome Biol..

[19] Hatzikotoulas K, Gilly A, Zeggini E (2014). Using population isolates in genetic association studies. Brief Funct. Genomics.

[20] Zeggini E (2014). Using genetically isolated populations to understand the genomic basis of disease. Genome Med..

[21] Pichler I (2009). Genetic structure in contemporary south Tyrolean isolated populations revealed by analysis of Y-chromosome, mtDNA and Alu polymorphisms. Hum. Biol..

[22] Esko T (2013). Genetic characterization of northeastern Italian population isolates in the context of broader European genetic diversity. Eur. J. Hum. Genet..

[23] Capocasa M (2014). Linguistic, geographic and genetic isolation: A collaborative study of Italian populations. J. Anthropol. Sci..

[24] Sarno S (2016). Shared language, diverging genetic histories: High-resolution analysis of Y-chromosome variability in Calabrian and Sicilian Arbereshe. Eur. J. Hum. Genet..

[25] Anagnostou P (2017). Overcoming the dichotomy between open and isolated populations using genomic data from a large European dataset. Sci. Rep..

[26] Anagnostou P (2019). Inter-individual genomic heterogeneity within European population isolates. PLoS ONE.

[27] Sarno S (2017). Ancient and recent admixture layers in Sicily and Southern Italy trace multiple migration routes along the Mediterranean. Sci. Rep..

[28] Pott, F.A. Altgriechisch in heutigen Kalabrien? In *Philologus*, 244–269 (Dieterich, 1856).

[29] Comparetti, D. *Saggi sui dialetti greci dell’Italia meridionale*. (Fratelli Nistri, 1866).

[30] Pellegrini, A. *Il dialetto greco-calabro di Bova *(Loescher, 1880).

[31] Alessio, G. Il sostrato latino nel lessico e nell’epo-toponomastica della Calabria meridionale, in *L’Italia Dialettale*, 111–190 (Simoncini, 1934).

[32] Battisti, C. Ancora sulla grecità in Calabria, in *Archivio storico per la Calabria e la Lucania,* 67–95 (Società Magna Grecia, 1933).

[33] Chatzidakis, G. N. *Einleitung in die neugriechischen Grammatik*. (Breikopf & Hartl, 1892).

[34] Rohlfs, G. *Scavi linguistici nella Magna Grecia*. (Congedo ed., 1932).

[35] Tsopanakis, A. G. Echi classici nel greco della Magna Grecia. In *Magna Grecia bizantina e tradizione classica: atti del XVII convegno di studi sulla Magna Grecia* (Ist. Storia Arch. Magna Grecia, 1977).

[36] Fanciullo, F. Latinità e grecità in Calabria. In *Storia della Calabria antica* (Gangemi ed., 2000).

[37] Trumper, J. *Geostoria linguistica della Calabria*. (Aracne ed., 2016).

[38] Mosino, F. *Minoranze etniche in Calabria e Basilicata*. (Di Mauro ed., 1988).

[39] Martino, P. L’Isola grecanica dell’Aspromonte. Aspetti sociolinguistici. In *Atti dell’XI Congresso Nazionale di Studi (Cagliari 27–30 maggio 1977)*, (ed. Leoni, F. A.) 305–341 (Bulzoni, 1980).

[40] Violi, F. *Storia della Calabria Greca con particolare riguardo all’odierna isola ellenofona*. (Kaleidon ed., 2006).

[41] Squillaci, M. O. When a language becomes old. The case of Calabrian Greek. In *Selected papers from the XV International Conference on Minority Languages* (University of Belgrade, 2017).

[42] Lao O (2008). Correlation between genetic and geographic structure in Europe. Curr. Biol..

[43] Novembre J (2008). Genes mirror geography within Europe. Nature.

[44] Chiang CWK (2018). Genomic history of the Sardinian population. Nat. Genet..

[45] Marcus JH (2020). Genetic history from the Middle Neolithic to present on the Mediterranean island of Sardinia. Nat. Commun..

[46] Palamara PF, Lencz T, Darvasi A, Peer I (2012). Length distributions of identity by descent reveal fine-scale demographic history. Am. J. Hum. Genet..

[47] Minuto, D. *Storia della gente in Calabria*. (Qualecultura, 2005).

[48] Tagliamonte, G. Le popolazioni indigene. In *Magna Grecia - Città greche di Magna Grecia e Sicilia* (eds. D’Andria, F. & Guzzo, P.) 20–28 (Istituto dell’Enciclopedia Treccani, 2012).

[49] Pouyet F, Aeschbacher S, Thiéry A, Excoffier L (2018). Background selection and biased gene conversion affect more than 95% of the human genome and bias demographic inferences. Elife..

[50] Huang S (2016). New thoughts on an old riddle: What determines genetic diversity within and between species?. Genomics.

[51] Pouyet, F. & Gilbert, K. J. Towards an improved understanding of molecular evolution: the relative roles of selection, drift, and everything in between. *arXiv.* Vol. 1909, 11490. ver.4 peer-reviewed and recommended by PCI Evolutionary Biology. https://arxiv.org/abs/1909.11490 (2020).

[52] Bernardi L (2012). Epidemiology and genetics of frontotemporal dementia: A door-to-door survey in southern Italy. Neurobiol. Aging.

[53] Anfossi M (2014). Identification of three novel LRRK2 mutations associated with Parkinson's disease in a Calabrian population. J. Alzheimers. Dis..

[54] Bernardi L (2014). Novel N-terminal domain mutation in prion protein detected in 2 patients diagnosed with frontotemporal lobar degeneration syndrome. Neurobiol. Aging.

[55] Conidi ME (2015). Homozygous carriers of APP A713T mutation in an autosomal dominant Alzheimer disease family. Neurology.

[56] Borrello L (2016). Angela R.: A familial Alzheimer's disease case in the days of Auguste D.. J. Neurol..

[57] Cupidi C, Laganà V, Smirne N, Bruni CA (2017). The role of historical medical archives in the genealogical rebuilding of large families affected by neurodegenerative diseases. J. Neurol. Neuromed..

[58] Maletta R (2018). Frequency of cardiovascular genetic risk factors in a calabrian population and their effects on dementia. J. Alzheimers Dis..

[59] Panoutsopoulou K (2014). Genetic characterization of Greek population isolates reveals strong genetic drift at missense and trait-associated variants. Nat. Commun..

[60] Chang CC, Chow CC, Tellier LC, Vattikuti S, Purcell S, Lee JJ (2015). Second-generation PLINK: Rising to the challenge of larger and richer datasets. Gigascience.

[61] Li JZ (2008). Worldwide human relationships inferred from genome-wide patterns of variation. Science.

[62] Lazaridis I (2014). Ancient human genomes suggest three ancestral populations for present-day Europeans. Nature.

[63] Haak W (2015). Massive migration from the steppe was a source for Indo-European languages in Europe. Nature.

[64] Mathieson I (2015). Genome-wide patterns of selection in 230 ancient Eurasians. Nature.

[65] Jones ER (2015). Upper Palaeolithic genomes reveal deep roots of modern Eurasians. Nat. Commun..

[66] Hofmanová Z (2016). Early farmers from across Europe directly descended from Neolithic Aegeans. Proc. Natl. Acad. Sci. USA.

[67] Lazaridis I (2016). Genomic insights into the origin of farming in the ancient Near East. Nature.

[68] Fu Q (2016). The genetic history of Ice Age Europe. Nature.

[69] Lazaridis I (2017). Genetic origins of the Minoans and Mycenaeans. Nature.

[70] Lipson M (2017). Parallel palaeogenomic transects reveal complex genetic history of early European farmers. Nature.

[71] Mathieson I (2018). The genomic history of southeastern Europe. Nature.

[72] Olalde I (2018). The Beaker phenomenon and the genomic transformation of northwest Europe. Nature.

[73] Patterson N, Price AL, Reich D (2006). Population structure and eigenanalysis. PLoS Genet..

[74] Alexander DH, Novembre J, Lange K (2009). Fast model-based estimation of ancestry in unrelated individuals. Genome Res..

[75] Pickrell JK, Pritchard JK (2012). Inference of population splits and mixtures from genome-wide allele frequency data. PLoS Genet..

[76] Reich D, Thangaraj K, Patterson N, Price AL, Singh L (2009). Reconstructing Indian population history. Nature.

[77] Patterson N (2012). Ancient admixture in human history. Genetics.

[78] Wang CC (2019). Ancient human genome-wide data from a 3000-year interval in the Caucasus corresponds with eco-geographic regions. Nat. Commun..

[79] Antonio ML (2019). Ancient Rome: A genetic crossroads of Europe and the Mediterranean. Science.

[80] Lawson DJ, Hellenthal G, Myers S, Falush D (2012). Inference of population structure using dense haplotype data. PLoS Genet..

[81] Delaneau O, Zagury JF, Marchini J (2013). Improved whole-chromosome phasing for disease and population genetic studies. Nat. Methods.

[82] Kirin M, McQuillan R, Franklin CS, Campbell H, McKeigue PM, Wilson JF (2010). Genomic runs of Homozygosity record population history and consanguinity. PLoS ONE.

[83] Pemberton TJ, Absher D, Feldman MW, Myers RM, Rosenberg NA, Li JZ (2012). Genomic patterns of homozygosity in worldwide human populations. Am. J. Hum. Genet..

[84] Browning BL, Browning SR (2011). A fast, powerful method for detecting identity by descent. Am. J. Hum. Genet..

[85] Ralph P, Coop G (2013). The geography of recent genetic ancestry across Europe. PLoS Biol..

[86] Moreno-Estrada A (2014). Human genetics. The genetics of Mexico recapitulates Native American substructure and affects biomedical traits. Science.

[87] Thomas PD (2006). Applications for protein sequence-function evolution data: mRNA/protein expression analysis and coding SNP scoring tools. Nucleic Acids Res..

[88] Mi H, Muruganujan A, Ebert D, Huang X, Thomas PD (2019). PANTHER version 14: More genomes, a new PANTHER GO-slim and improvements in enrichment analysis tools. Nucleic Acids Res..

[89] Smedley D (2015). The BioMart community portal: An innovative alternative to large, centralized data repositories. Nucleic Acids Res..

[90] Yates B, Braschi B, Gray KA, Seal RL, Tweedie S, Bruford EA (2017). Genenames.org: The HGNC and VGNC resources in 2017. Nucleic Acids Res..

[91] Yates AD, Achuthan P, Akanni W, Allen J, Allen J, Alvarez-Jarreta J (2020). Ensembl 2020. Nucleic Acids Res..

[92] Loecher M, Ropkins K (2015). RgoogleMaps and loa: Unleashing R graphics power on map tiles. J. Stat. Softw..

