# Supplementary material for: Genetic history of Calabrian Greeks reveals ancient events and long term isolation in the Aspromonte area of Southern Italy
Source: Sci Rep. 2021 Feb 4;11:3045. doi: 10.1038/s41598-021-82591-9 (PMC7862261; doi:10.1038/s41598-021-82591-9)
Supplement: Supplementary file 1 — Supplementary Information 1. [file 41598_2021_82591_MOESM1_ESM.pdf]

Genetic history of Calabrian Greeks reveals ancient events and long term isolation in the Aspromonte area of Southern Italy

Stefania Sarno[1,^,*], Rosalba Petrilli[1,^], Paolo Abondio[1,^], Andrea De Giovanni[1,2], Alessio Boattini[1], Marco Sazzini[1,3], Sara De Fanti[1,3], Elisabetta Cilli[2], Graziella Ciani[1], Davide Gentilini[4,5], Davide Pettener[1], Giovanni Romeo[6,7], Cristina Giuliani[1,#], Donata Luiselli[2,#,*].

[1]Department of Biological, Geological and Environmental Sciences, University of Bologna, Bologna, Italy

[2]Department of Cultural Heritage, University of Bologna, Ravenna, Italy

[3]Interdepartmental Centre Alma Mater Research Institute on Global Challenges and Climate Change, University of Bologna, Bologna, Italy

[4]Department of Brain and Behavioral Sciences, University of Pavia, Pavia, Italy

[5]Italian Auxologic Institute IRCCS, Cusano Milanino, Milan, Italy

[6]Medical Genetics Unit, Sant'Orsola-Malpighi University Hospital, Bologna, Italy.

[7]European School of Genetic Medicine, Italy

[^] These authors contributed equally to this work

[#] These authors share co-senior authorship

[*] Corresponding authors: donata.luiselli@unibo.it; stefania.sarno2@unibo.it

Supplementary Figures

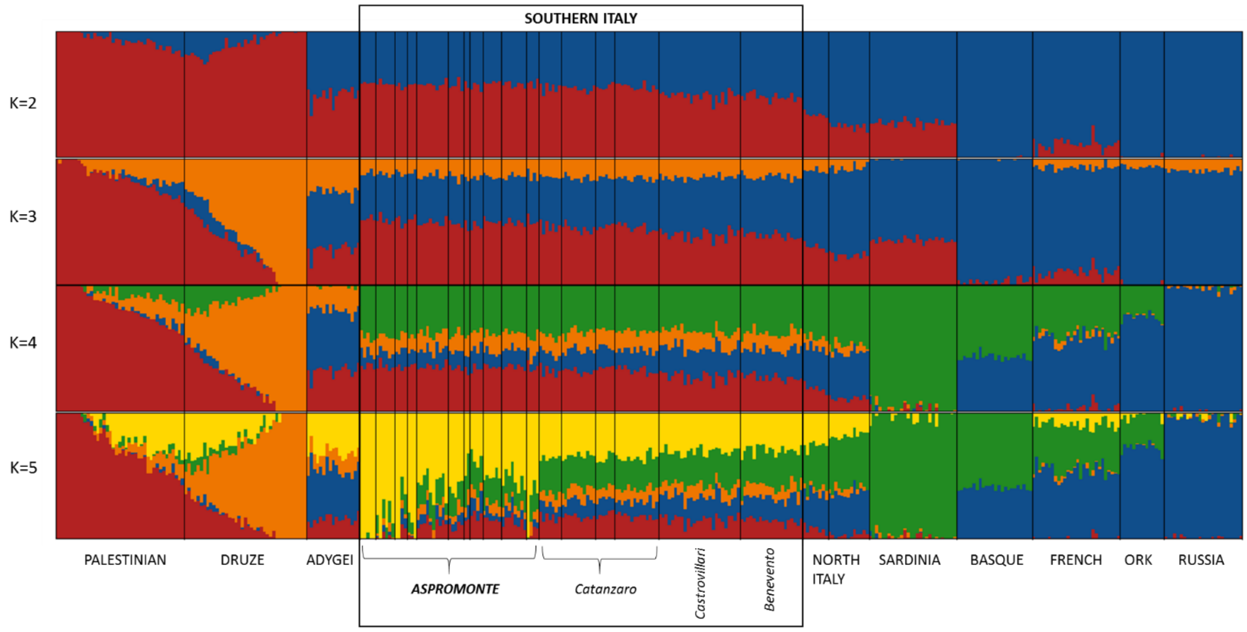

Supplementary Figure S1. ADMIXTURE analysis performed on the modern extended dataset. At any K, from 2 (top) through 5 (bottom), each of the 379 individuals is represented by a vertical (100%) column of genetic component probabilities, colored according to the K reconstructed ancestral populations. Individuals are grouped and labelled at population level.

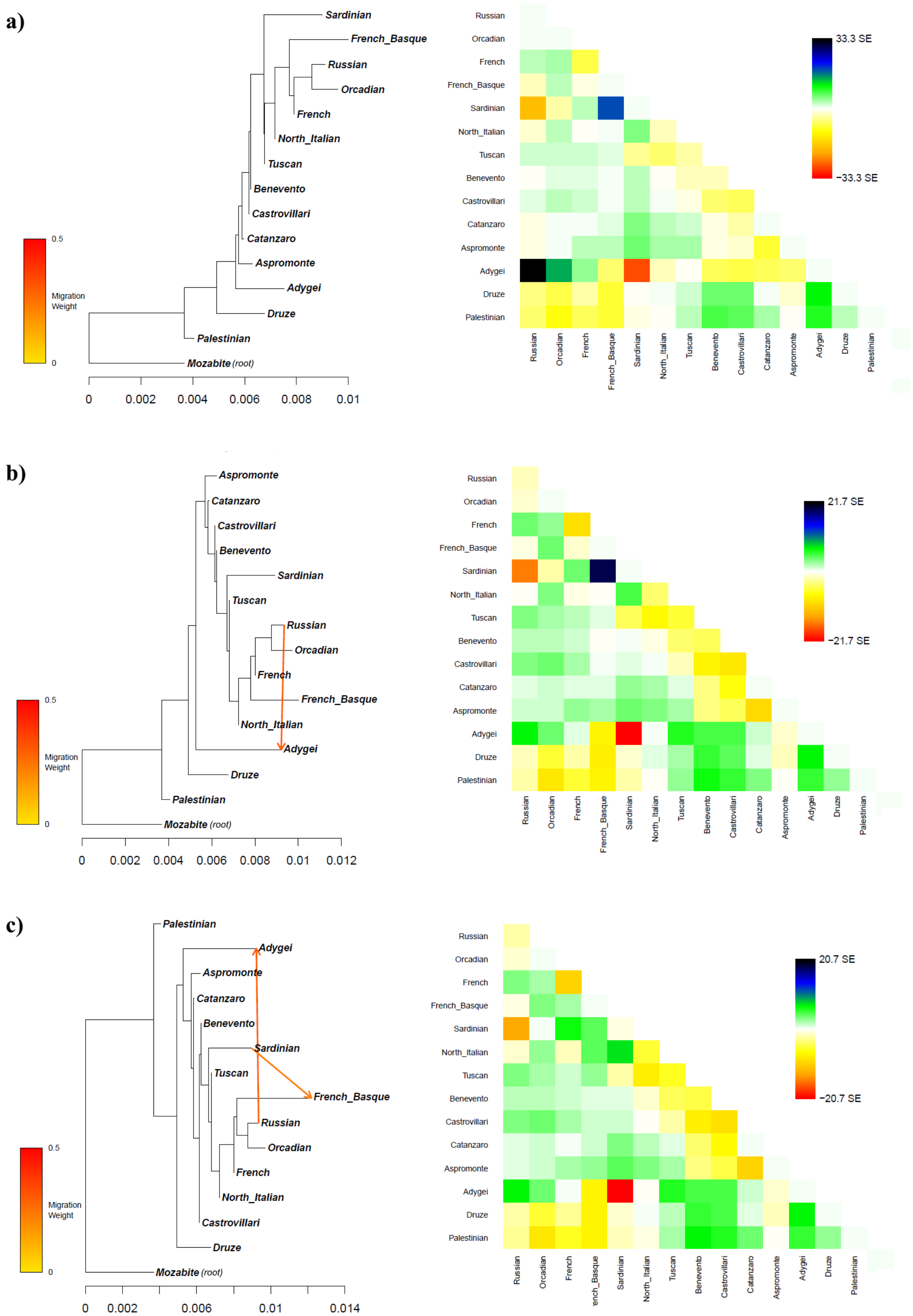
a)
Sardinian
French_Basque
Russian
Orcadian
French
North_Italian
Tuscan
Benevento
Castrovillari
Catanzaro
Aspromonte
Adygei
Druze
Palestinian
Mozabite (root)
0.5
Migration Weight
0
0 0.002 0.004 0.006 0.008 0.01
Russian
Orcadian
French
French_Basque
Sardinian
North_Italian
Tuscan
Benevento
Castrovillari
Catanzaro
Aspromonte
Adygei
Druze
Palestinian
33.3 SE
−33.3 SE
b)
Aspromonte
Catanzaro
Castrovillari
Benevento
Sardinian
Tuscan
Russian
Orcadian
French
French_Basque
North_Italian
Adygei
Druze
Palestinian
Mozabite (root)
0.5
Migration Weight
0
0 0.002 0.004 0.006 0.008 0.01 0.012
21.7 SE
−21.7 SE
c)
Palestinian
Adygei
Aspromonte
Catanzaro
Benevento
Sardinian
Tuscan
French_Basque
Russian
Orcadian
French
North_Italian
Castrovillari
Druze
Mozabite (root)
0.5
Migration Weight
0
0 0.002 0.006 0.01 0.014
20.7 SE
−20.7 SE

d)

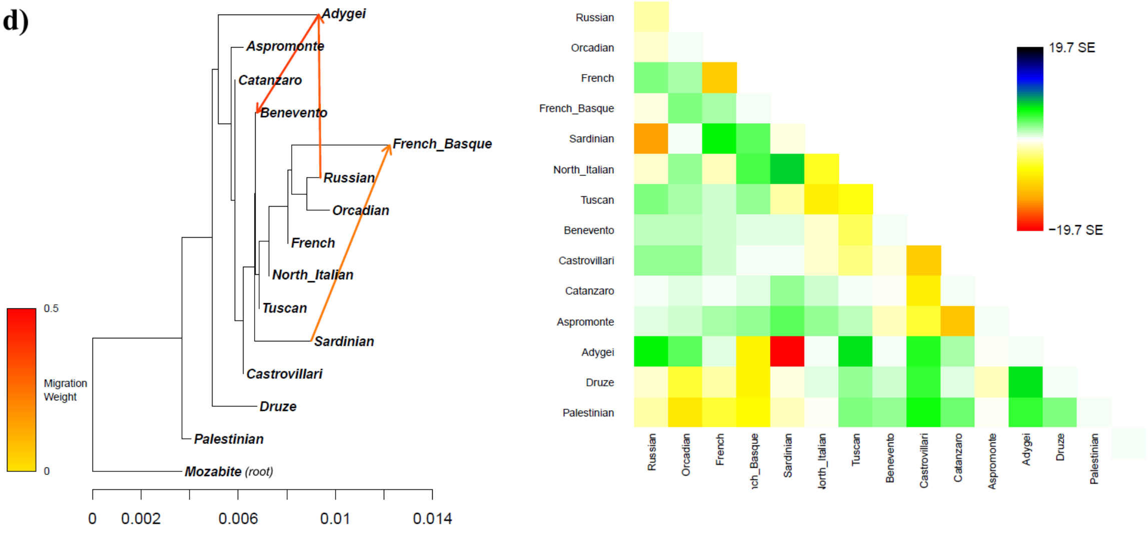

Adygei
Aspromonte
Catanzaro
Benevento
French_Basque
Russian
Orcadian
French
North_Italian
Tuscan
Sardinian
Castrovillari
Druze
Palestinian
Mozabite (root)
0.5
Migration Weight
0
0 0.002 0.006 0.01 0.014
19.7 SE
−19.7 SE
Russian
Orcadian
French
French_Basque
Sardinian
North_Italian
Tuscan
Benevento
Castrovillari
Catanzaro
Aspromonte
Adygei
Druze
Palestinian

e)

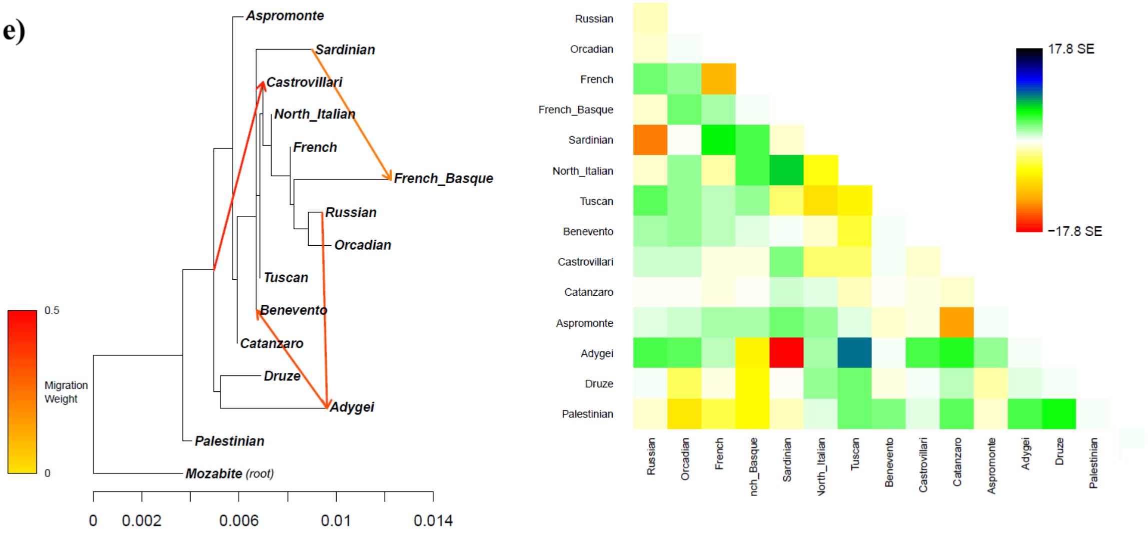

Aspromonte
Sardinian
Castrovillari
North_Italian
French
French_Basque
Russian
Orcadian
Tuscan
Benevento
Catanzaro
Druze
Adygei
Palestinian
Mozabite (root)
0.5
Migration Weight
0
0 0.002 0.006 0.01 0.014
17.8 SE
−17.8 SE
Russian
Orcadian
French
French_Basque
Sardinian
North_Italian
Tuscan
Benevento
Castrovillari
Catanzaro
Aspromonte
Adygei
Druze
Palestinian

f)

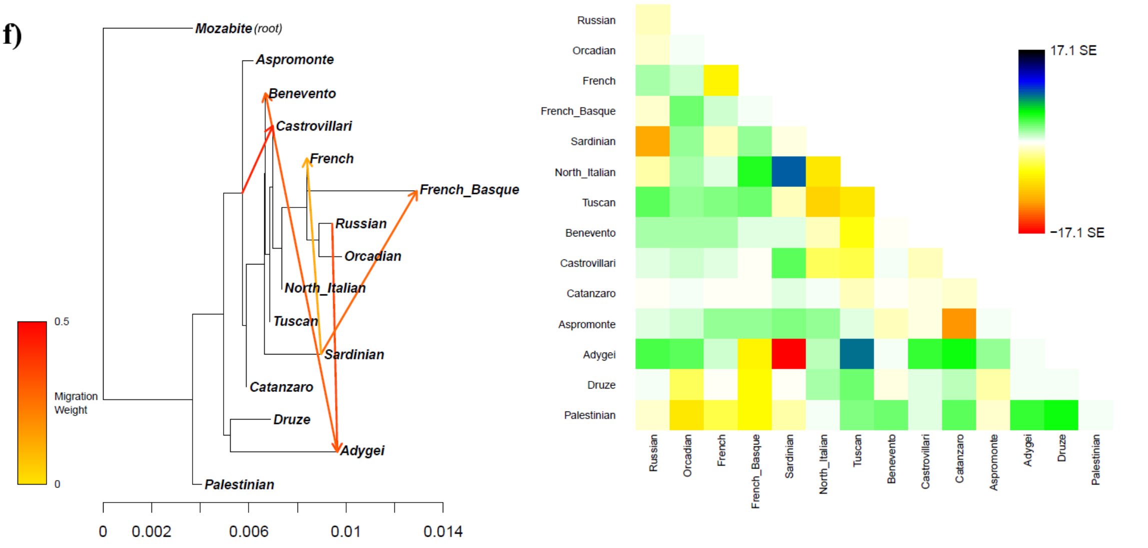

Mozabite (root)
Aspromonte
Benevento
Castrovillari
French
French_Basque
Russian
Orcadian
North_Italian
Tuscan
Sardinian
Catanzaro
Druze
Adygei
Palestinian
0.5
Migration Weight
0
0 0.002 0.006 0.01 0.014
17.1 SE
−17.1 SE
Russian
Orcadian
French
French_Basque
Sardinian
North_Italian
Tuscan
Benevento
Castrovillari
Catanzaro
Aspromonte
Adygei
Druze
Palestinian

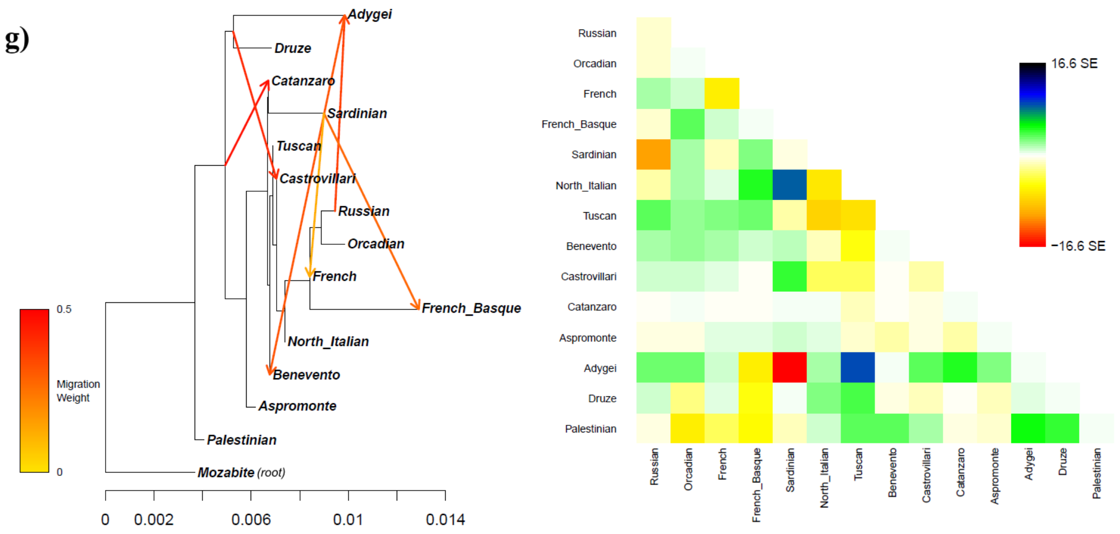

Supplementary Figure S2. TreeMix graphs describing the splitting patterns observed in the populations of the modern extended dataset. Trees were constructed by allowing for *m=0* through *m=6* migrations (panels from *a* to *g*). The length of the branches is proportional to the genetic drift experienced by each population. Heatmaps on the right of each plot show the matrix of residuals associated to the performed TreeMix models.

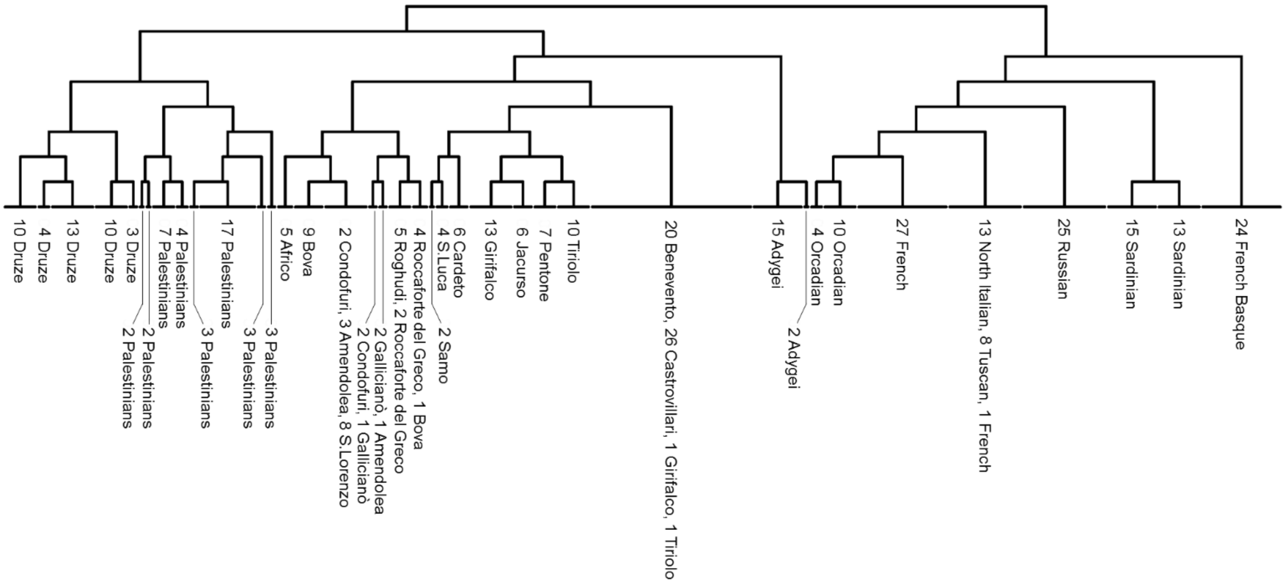

Supplementary Figure S3. Dendrogram of the fineSTRUCTURE hierarchical clustering. The labels detail the population name and number of individuals from each population belonging to the identified clusters.

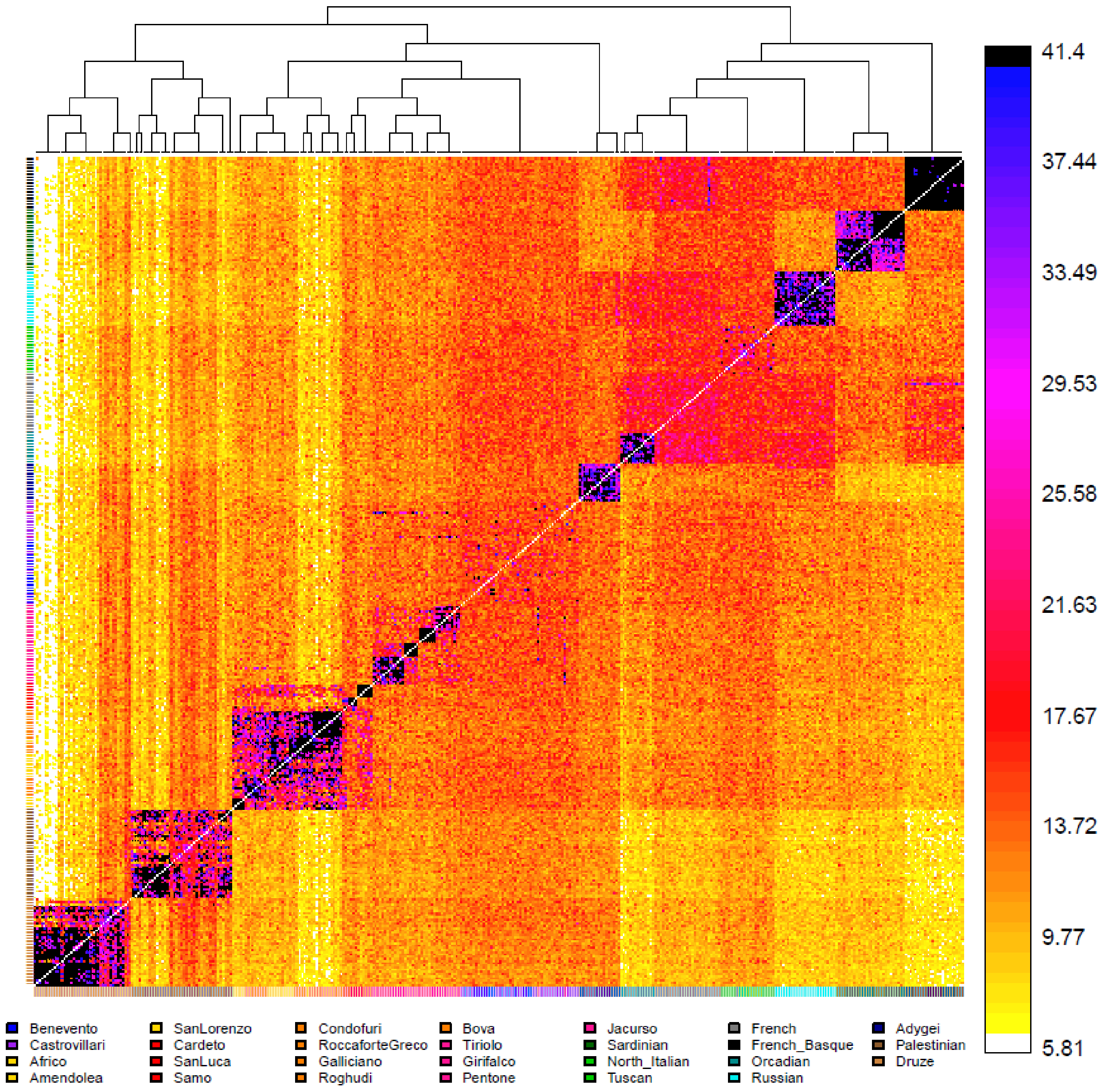

Supplementary Figure S4. FineSTRUCTURE hierarchical clustering (top) and chunk-lengths heatmap matrix (bottom) of total length of haplotypes shared between the pairs of individuals of the modern extended dataset.

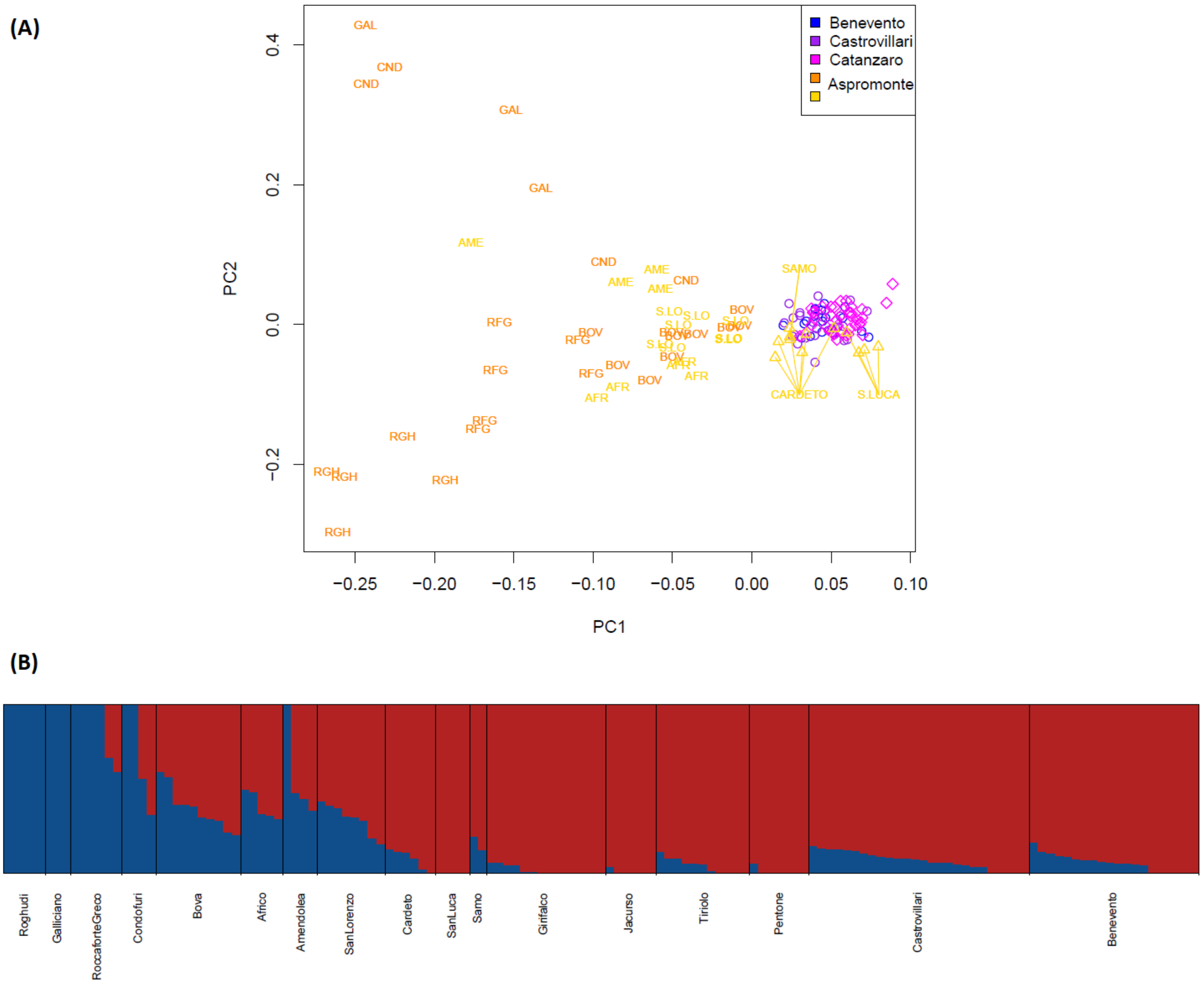

Supplementary Figure S5. Population structure analyses performed on the pruned Southern Italian local dataset. **(a)** Scatter plot of the first and second PCs computed on the 141 individuals from the Southern Italian populations newly-analyzed in the present study. Individuals are color coded according to their province of origin as in the legend at the top right: Benevento (*blue*); Castrovillari (*purple*); Catanzaro (*magenta*); previously collected samples from Reggio Calabria (*orange*); newly collected samples from Reggio Calabria (*gold*). For a further detail, individuals from the Aspromonte mountain area are labelled based on their corresponding villages. **(b)** Results of unsupervised ADMIXTURE cluster-based analysis for K = 2 (i.e. the best-fit model according to CV-errors). Each of the 141 Southern Italian individuals is represented by a vertical (100%) column of genetic component probabilities, colored based on the K=2 reconstructed ancestral populations. Individuals are grouped and labelled at population level.

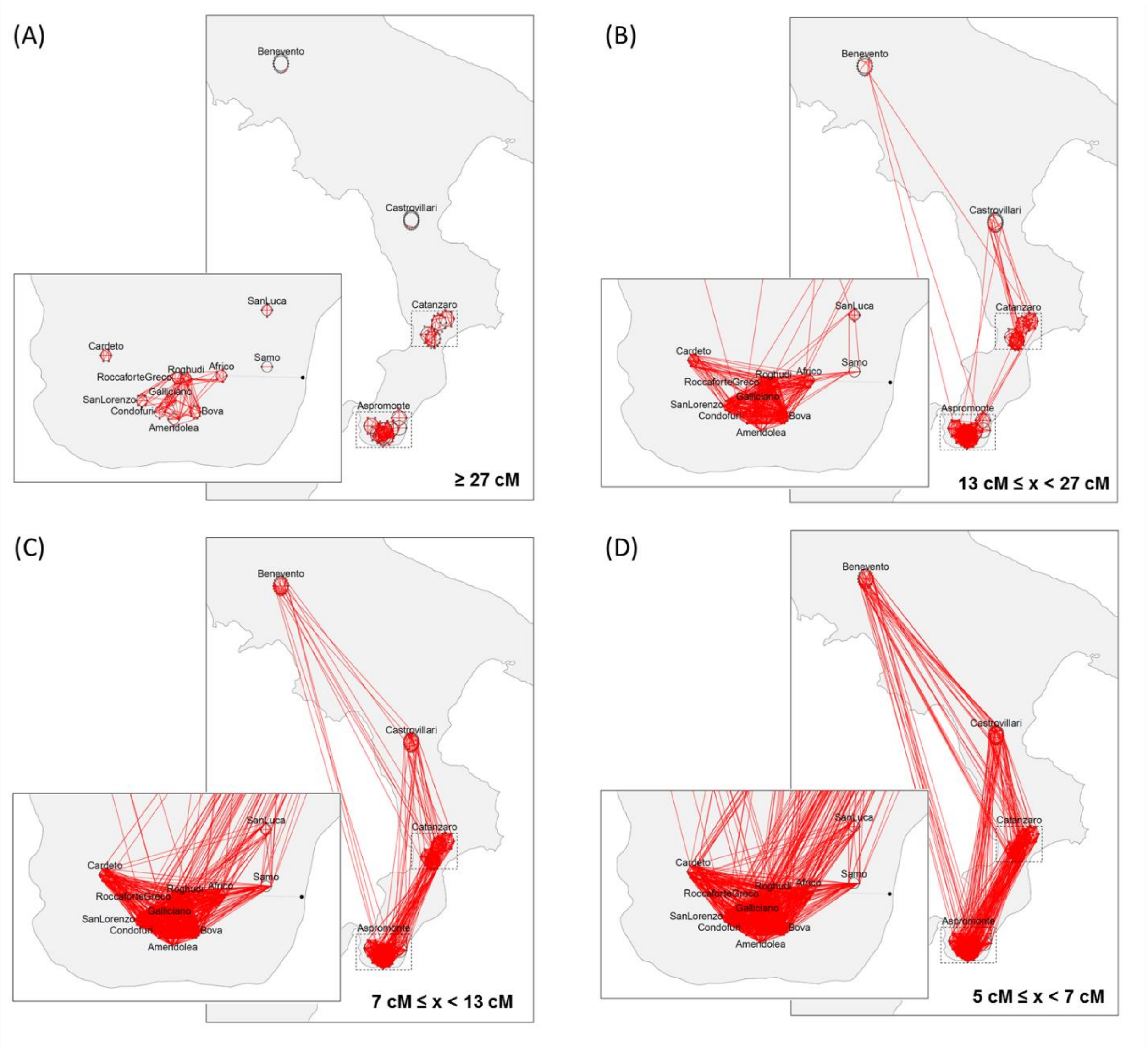

Supplementary Figure S6. Patterns of relatedness within and between Southern Italian populations measured by the total amount of segments shared identical-by-descent (IBD) between pairs of individuals at different classes of length. Each dot represents an individual and each line denotes a sharing match between two individuals. To minimize false positive IBD matches, a minimum length of 5 cM was required to be considered in the plot. In order to provide geographic context, individuals from the same population are displayed around the position that approximate the location of the sampled population. Each plot shows the network of cumulative connections resulting for each bin class of length. The magnification within each plot is aimed at zooming on the connections within the Aspromonte mountain area.

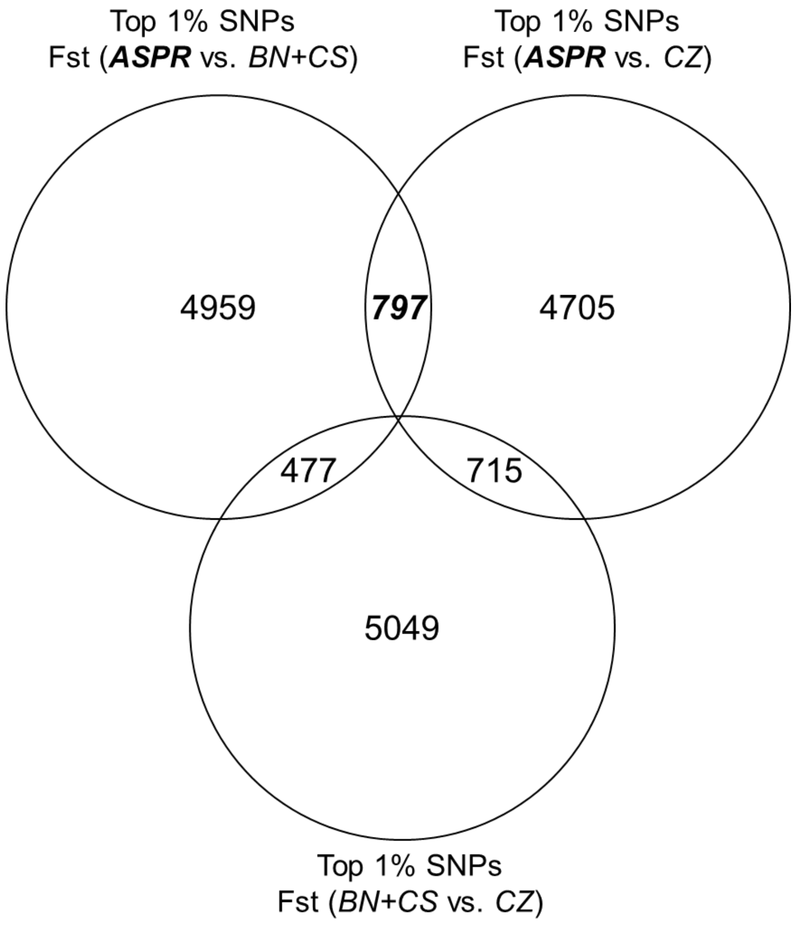

Supplementary Figure S7. Venn diagrams showing the number of loci scoring in the top 1% FST values of the comparisons between Aspromonte (*ASPR*) vs. Benevento+Castrovillari (*BN+CS*), Aspromonte (*ASPR*) vs. Catanzaro (*CZ*), and Benevento+Castrovillari (*BN+CS*) vs. Catanzaro (*CZ*) clusters identified by FineSTRUCTURE. The 797 SNPs differentiating the *ASPR* cluster from both *BN+CS* and *CZ* ones were highlighted in bold and listed in Supplementary Table S4.

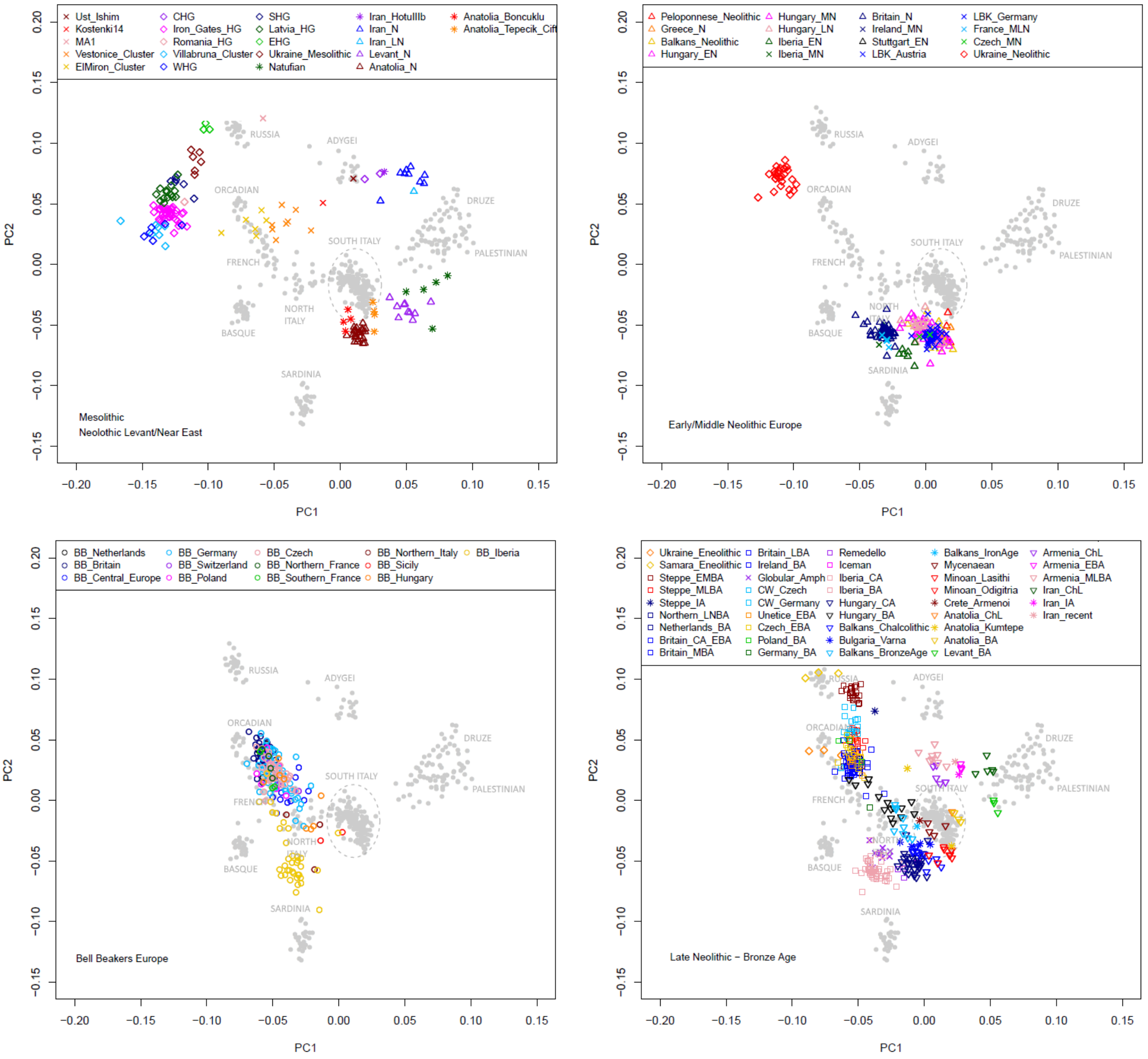

Supplementary Figure S8. Principal component analysis performed by projecting ancient samples onto the space defined by modern individuals (in gray). Scatterplot of the first and second PCs for **(a)** Mesolithic samples and ancient individuals from the Neolithic of the Levant and the Near East; **(b)** ancient samples from the Early and Middle Neolithic of Europe; **(c)** ancient individuals associated to the Bell Beakers culture; and **(d)** samples from the Late Neolithic and the Bronze Age. Ancient individuals are labelled and symbol-coded according to their associated culture, as reported in the legend at the top of each plot. The position of newly-analyzed Southern Italian populations in the PCA space defined by modern individuals is indicated by the dashed gray circle.

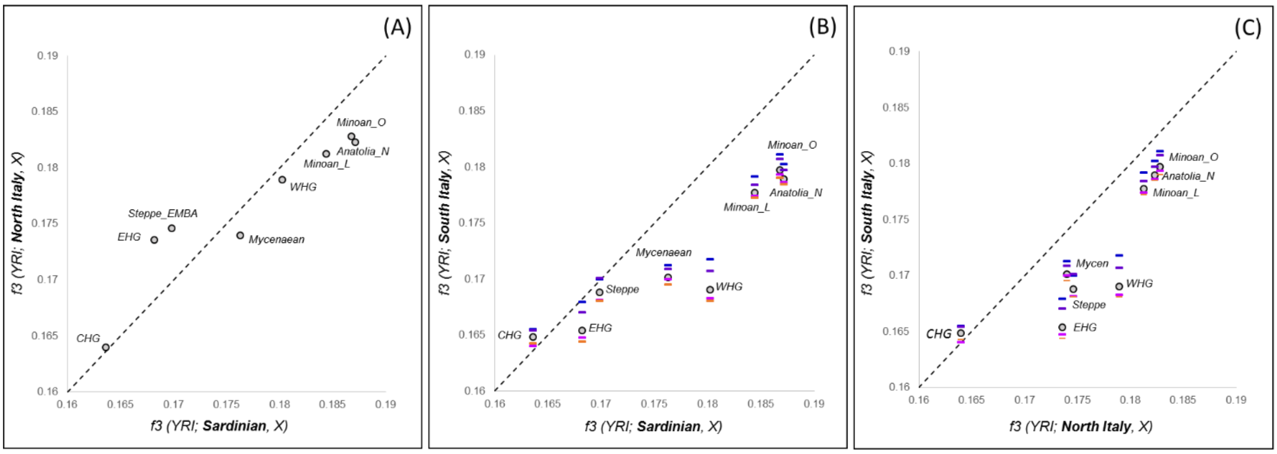

Supplementary Figure S9. Three-population *f3* statistics with Yoruba as outgroup in the form of *f3 (YRI; Test, A)* comparing the extent of shared genetic drift between **(a)** Sardinia (x-axis) or Northern Italy (y-axis) as *Test* and the ancient selected groups as *A*; **(b)** Sardinia (x-axis) or Southern Italian populations (y-axis) as *Test* and ancient selected groups as *A*; **(c)** Northern Italy (x-axis) or Southern Italian populations (y-axis) as *Test* and ancient selected groups as *A*. The dashed line represents the x = y straight line. The labels of the considered ancient populations are reported in the plot. For comparison involving Southern Italy, the *f3-outgroup* statistics was computed both as a whole (gray dot) and by considering the single populations separately (Benevento: blue dash, Castrovillari: purple dash, Catanzaro: magenta dash, Aspromonte area: orange dash).

a)

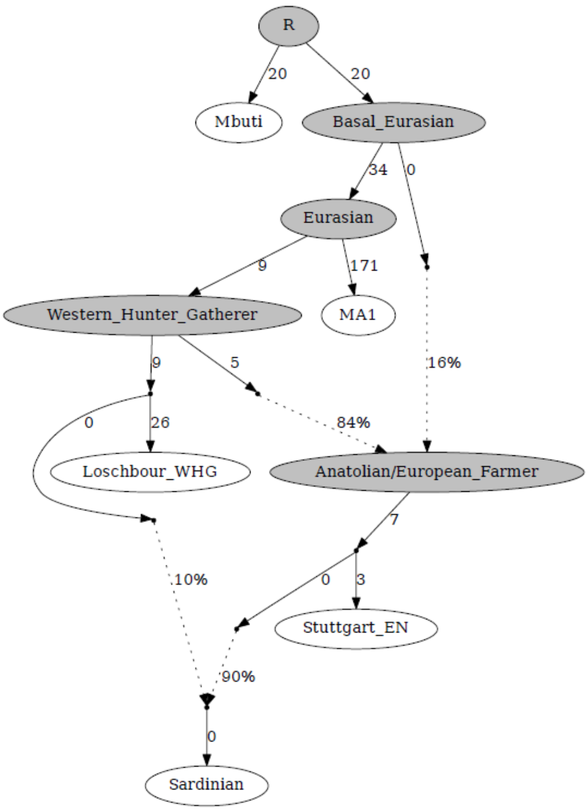
R
20
20
Mbuti
Basal_Eurasian
34
0
Eurasian
9
171
MA1
Western_Hunter_Gatherer
16%
9
5
0
26
84%
Loschbour_WHG
Anatolian/European_Farmer
7
10%
0
3
Stuttgart_EN
90%
0
Sardinian

b)

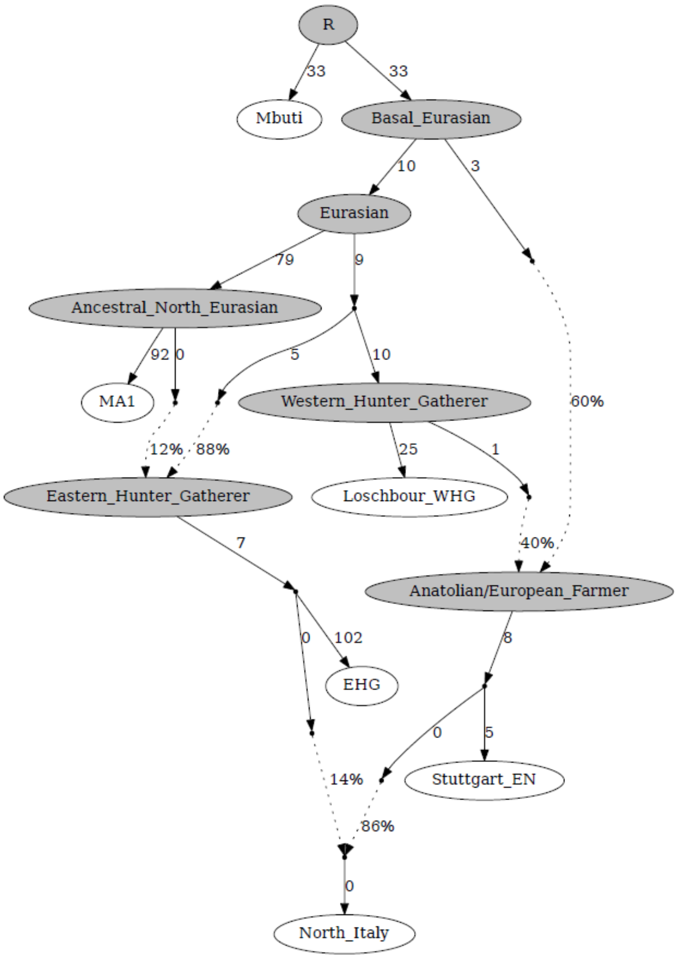
R
33
33
Mbuti
Basal_Eurasian
10
3
Eurasian
79
9
Ancestral_North_Eurasian
92
0
5
10
MA1
Western_Hunter_Gatherer
60%
12%
88%
25
1
Eastern_Hunter_Gatherer
Loschbour_WHG
7
40%
Anatolian/European_Farmer
0
102
8
EHG
0
5
14%
Stuttgart_EN
86%
0
North_Italy

c)

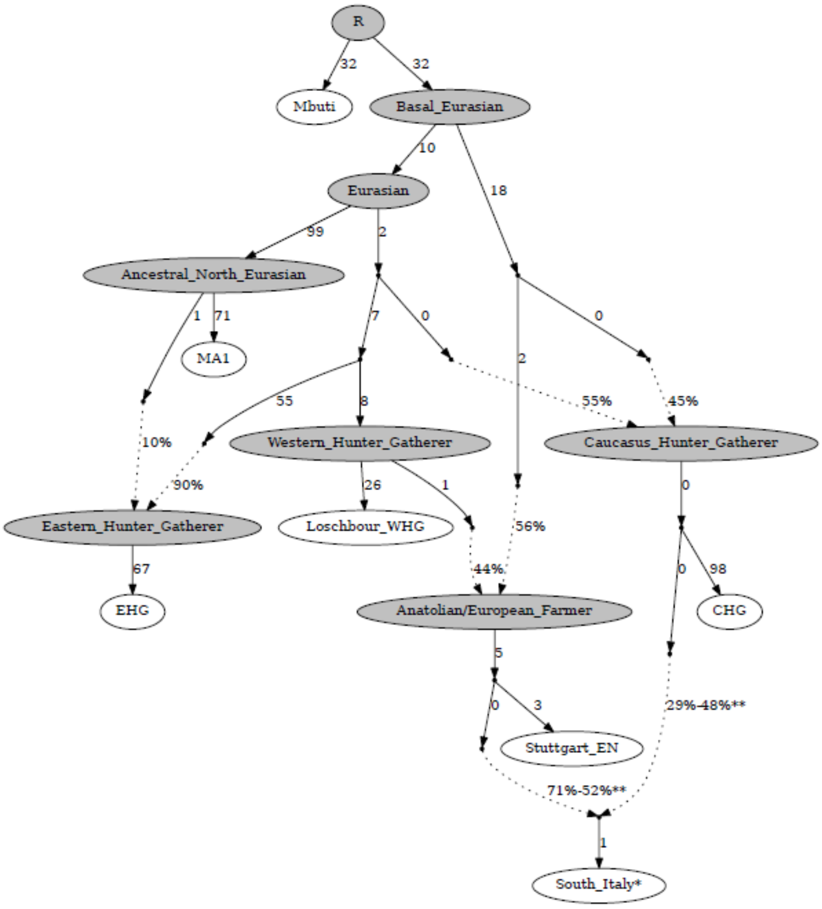

* Schematic summary of Admixture Graphs obtained testing in turn the four Southern Italian groups (i.e. Benevento, Castrovillari, Catanzaro and Aspromonte).
**Ranges of admixture proportions represent the min and max values observed, while details about the obtained population-specific values are reported below:

Benevento:	71% - 29%
Castrovillari:	65% - 35%
Catanzaro:	56% - 44%
Aspromonte:	52% - 48%

Supplementary Figure S10. Admixture Graph fitting modelling obtained with *qpGraph*. **(a)** The model in which Sardinian are admixed deriving ancestry from Early Farmer and West European Hunter-Gatherer related lineages is a fit to the data in the sense that there are no f-statistics more than |Z|>3 different between model and expectation (Z-score of the worst f-statistic = 0.193). **(b)** We added an admixture event from an EHG-related lineage to North Italy, which is a fit to the data in the sense that no f-statistics more than |Z|>3 different between model and expectation have been observed for North Italy (Z-score of the worst f-statistic = -1.086). This model is not a fit to the data for Southern Italians since in that case there are outlier f-statistics (worst Z-score = -5.180). **(c)** The model fitting Southern Italian populations as deriving ancestry from a CHG-related lineage instead fit the data in the sense that for this model there are no f-statistics more than |Z|>3 different between model and expectation (Z-score of the worst f-statistic = 2.286). Dotted lines represent the two-way admixture events tested and the percentages of ancestry on each line denote the proportions of admixture relative to the two admixing lineages.

Supplementary Tables

Supplementary Tables S1-S8 are included as a separated Excel data file.

Supplementary Table S1. List of Southern Italian populations newly-genotyped in the present study and of reference Euro-Mediterranean groups used for comparisons.

Supplementary Table S2. *f3*-testing of admixture. For each Southern Italian population (*Target*) we computed *f3*-statistics using all possible pairs of other comparison populations as sources (*Source1-Source2*). Population pairs for which *f3* produced significantly negative z-scores (i.e. Z<-3) are highlighted in red.

Supplementary Table S3. Inbreeding coefficient (*Fin*) and genome-wide homozygosity (*Fhom*) in the considered Southern Italian population groups.

Supplementary Table S4. List of the 797 top 1% Fst SNPs differentiating the Aspromonte cluster (*ASPR*) from both the Benevento+Castrovillari (*BN+CS*) and Catanzaro (*CZ*) ones.

Supplementary Table S5. List of genes covered by the top 1% Fst SNPs differentiating the *ASPR* group from both *BN+CS* and *CZ* clusters.

Supplementary Table S6. Enrichment analysis on the list of top genes showing the most significantly enriched Gene Ontology (GO) terms.

Supplementary Table S7. List of ancient samples included in the comparisons with modern populations.

Supplementary Table S8. Results of the four-population scenarios modelled with *qpAdmix* for the Italian populations using ancient putative sources.
